# Effect of Single Dose of Antimicrobial Administration at Birth on Fecal Microbiota Development and Prevalence of Antimicrobial Resistance Genes in Piglets

**DOI:** 10.3389/fmicb.2019.01414

**Published:** 2019-06-19

**Authors:** Mohamed Zeineldin, Ameer Megahed, Brandi Burton, Benjamin Blair, Brian Aldridge, James F. Lowe

**Affiliations:** ^1^Integrated Food Animal Management Systems, Department of Veterinary Clinical Medicine, College of Veterinary Medicine, University of Illinois at Urbana–Champaign, Champaign, IL, United States; ^2^Department of Animal Medicine, College of Veterinary Medicine, Benha University, Benha, Egypt; ^3^Infectious Genomic of One Health, Carl R. Woese Institute for Genomic Biology, University of Illinois at Urbana–Champaign, Champaign, IL, United States

**Keywords:** antimicrobial, microbiota, neonatal piglets, resistance genes, fecal

## Abstract

Optimization of antimicrobial use in swine management systems requires full understanding of antimicrobial-induced changes on the developmental dynamics of gut microbiota and the prevalence of antimicrobial resistance genes (ARGs). The purpose of this study was to evaluate the impacts of early life antimicrobial intervention on fecal microbiota development, and prevalence of selected ARGs (*ermB*, *tetO*, *tetW*, *tetC*, *sulI*, *sulII*, and *blaC*_TX–M_) in neonatal piglets. A total of 48 litters were randomly allocated into one of six treatment groups soon after birth. Treatments were as follows: control (CONT), ceftiofur crystalline free acid (CCFA), ceftiofur hydrochloride (CHC), oxytetracycline (OTC), procaine penicillin G (PPG), and tulathromycin (TUL). Fecal swabs were collected from piglets at days 0 (prior to treatment), 5, 10, 15, and 20 post treatment. Sequencing analysis of the V3-V4 hypervariable region of the 16S rRNA gene and selected ARGs were performed using the Illumina Miseq platform. Our results showed that, while early life antimicrobial prophylaxis had no effect on individual weight gain, or mortality, it was associated with minor shifts in the composition of fecal microbiota and noticeable changes in the abundance of selected ARGs. Unifrac distance metrics revealed that the microbial communities of the piglets that received different treatments (CCFA, CHC, OTC, PPG, and TUL) did not cluster distinctly from CONT piglets. Compared to CONT group, PPG-treated piglets exhibited a significant increase in the relative abundance of *ermB* and *tetW* at day 20 of life. Tulathromycin treatment also resulted in a significant increase in the abundance of *tetW* at days 10 and 20, and *ermB* at day 20. Collectively, these results demonstrate that the shifts in fecal microbiota structure caused by perinatal antimicrobial intervention are modest and limited to particular groups of microbial taxa. However, early life PPG and TUL intervention could promote the selection of ARGs in herds. While additional investigations are required to explore the consistency of these findings across larger populations, these results could open the door to new perspectives on the utility of early life antimicrobial administration to healthy neonates in swine management systems.

## Introduction

The widespread use of injectable antimicrobials in the treatment and prevention of human and animal diseases continues to rise globally (MacKie et al., 2006). Numerous concerns related to human and animal health have been raised regarding the long-term sequelae of this trend, including emergence of antibiotic-resistant bacteria, dissemination of ARGs into the environment, perturbations of the gut microbiota-ecosystem and increased risk of diseases (Chee-Sanford et al., 2001; Hoelzer et al., 2017). Antimicrobial resistance develops when the administered antimicrobial eliminates susceptible microorganisms but leaves behind resistant strains that continue to grow and multiply (Wegener, 2003). These resistant bacteria transmit their genetic resistance characteristics to their progeny through vertical evolution, or to other bacterial species through horizontal evolution (Holmes et al., 2016). Recently, several lines of evidence indicate that extensive use and misuse of existing antimicrobials increases the numbers of ARG copies and risk of their spread among commensal bacterial population (Roca et al., 2015; Czaplewski et al., 2016; Zeineldin et al., 2019a).

Traditionally, the majority of studies evaluating the effect of antimicrobial administration on emergence of antibiotic resistant bacteria and ARGs have focused on pathogenic organisms using culture-based methods (Thanner et al., 2016). While this approach has enhanced our understanding of the nature of antimicrobial resistance in a single class of organisms, it is limited in its ecosystem-level application. Advancements in culture independent techniques such as next generation sequencing have allowed for the determination of microbial diversity in several animal biogeographic niches and have helped in the assessment of antimicrobial resistance determinants at the microbial ecosystem-level (Zhao et al., 2017; Zeineldin et al., 2019b).

Immediately after birth, the swine gastrointestinal tract is colonized by a complex microbial ecosystem, that plays a crucial role in the intestinal configuration, immune system maturation, and host gene expression (Zhang et al., 2016; Zeineldin et al., 2019c). During this stage, the microbial ecosystem is unstable and highly susceptible to various environmental factors, including antimicrobial administration, dietary intervention and stress exposure (Schokker et al., 2014). Given the instability of microbiota at this phase, the microbial population has the potential to disseminate and transfer ARGs, which could have significant effects on the development of metabolic and immune disorders (Gibson et al., 2015; Su et al., 2017). In intensive swine management systems, newborn piglets are frequently administered antimicrobials to prevent outbreaks of infectious diseases; however, the effects of early life antimicrobial prophylaxis on the emergence of ARGs and its connection with the gut microbial community in piglets are poorly understood. Recently, a study of early life antimicrobial intervention showed long-lasting impacts on the gastrointestinal microbial diversity and composition in newborn piglets (Schokker et al., 2014). In our previous study, we explored the change in the fecal microbiota of 8-weeks-old piglets in response to parenteral antimicrobial administration and we found that the fecal microbiota showed antimicrobial-specific variation in both duration and extent (Zeineldin et al., 2018a). To gain further insight into the swine gut ecosystem and to find alternatives to antimicrobials, it is crucial to understand the developmental dynamics of the gut microbiota and prevalence of ARGs in response to perinatal antimicrobial administration in piglets. Consequently, the aim of this study was to investigate the short-term impact of commonly used antimicrobials during early life on the developmental dynamics of the fecal microbiota, and relative abundance of selected ARGs (*ermB, sulI, sulII, tetC, tetO, tetW*, and *blaCTX-M*) in suckling piglets using high-throughput sequencing analysis.

## Materials and Methods

### Ethics Statement

This study was conducted in compliance with the recommendations of the guidelines for the care and use of animals of University of Illinois at Urbana–Champaign. The protocol was approved by the Ethical Committee for Institutional Animal Use and Care of the University of Illinois at Urbana–Champaign.

### Experimental Design and Samples Collection

The experiment was conducted in a commercial swine farm in the Midwestern US with consent from the facility owner. A total of 48 l were used in this study based on a randomized complete block design with farrowing day and dam parity group as blocks. Approximately five days before farrowing, the pregnant sows were transferred to a farrowing pen and kept there until the end of the experiment. Sows were fed a standard lactation diet, provided *ad libitum* access via an automatic dry feeding system, and were given *ad libitum* access to water from a nipple drinker. Directly after birth, litters were randomly assigned into one of six groups (*n* = 8 per group); control (CONT), ceftiofur crystalline free acid (CCFA), ceftiofur hydrochloride (CHC), oxytetracycline (OTC), procaine penicillin G (PGP), and tulathromycin (TUL). Littermates were used to minimize differences arising from maternal microbiota. After farrowing (day 0), all piglets were ear tagged and treatments were applied. All piglets in a litter were assigned to a single treatment group. The dosage schedule for each treatment group was as follow; CONT (saline 1cc IM), CCFA (5.0 mg /kg of body weight IM), CHC (5 mg/kg of body weight IM), OTC (22 mg/kg of body weight IM), PPG (33,000 units/kg of body weight) and TUL (2.5 mg/kg of body weight IM). CCFA and CHC are third-generation cephalosporins with a broad-spectrum activity against both Gram-positive and Gram-negative bacteria (Chander et al., 2011). OTC is a tetracycline antibiotic that also directly targets both Gram-positive and Gram-negative bacteria (Chopra and Roberts, 2001). PGP is one of the beta-lactam antibiotics that targets Gram-positive and Gram-negative bacteria (Ranheim et al., 2002). TUL is one of macrolide antibiotics that inhibit bacterial essential protein biosynthesis of both Gram-positive and Gram-negative bacteria (Schokker et al., 2014). The antimicrobial classes in this study are considered the most popular approved antibiotics used in the swine industry for the control and treatment of swine diseases (Schwarz et al., 2001).

The treated piglets were housed in a conventional farrowing pen that was approximately 1.9 m × 2.6 m where the sow was confined so that she could not turn around, and the sidewall penning for the piglets was solid to prevent contact between litters. All piglets were allowed to suckle colostrum and piglets were not added to the birth litter (some were removed prior to treatment if there were more pigs than available mammary glands). The antimicrobial dosages and routes of administration were based on the manufacturer label instructions. The piglet’s tails were not docked, and teeth were not clipped. All piglets were weighed individually at days 0 and 20 of life, and dead piglets were recorded throughout the study. Deep fecal swabs (Pur-Wraps^®^, Puritan Medical Products, Guilford, ME, United States) were collected immediately prior to treatment (day 0), and again on days 5, 10, 15, and 20 after dosing. The fecal swabs were snap-frozen in sterile containers and transported to the laboratory on the same day. Samples were kept at −80°C pending further processing.

### Extraction of Genomic DNA and Illumina Sequencing

Four clinically healthy piglets from each group (CONT, CCFA, CHC, OTC, PPG, and TUL) at the different sampling days (0, 5, 10, 15, and 20) were selected for the microbiota analysis. Negative control samples were also obtained from cotton swabs and extraction kit reagents. In a decontaminated sterile environment, microbial DNA was extracted from all selected samples using commercially available kits (MO BIO Laboratories, Inc., Carlsbad, CA, United States) (Zeineldin et al., 2017b, 2018b). Briefly, the swabs were mixed with 750 μl of Bead Solution (MO BIO Laboratories, Inc.), and bead beating was carried out in Bullet Blender 24 Gold tube holder machine (Next Advance, Inc., Averill Park, NY, United States) for 10 min. Then the extraction process was completed according to the manufacturer’s manual. The concentration and integrity of DNA were assessed using a Nanodrop^TM^ spectrophotometer (NanoDrop Technologies, Rockland, DE, United States), and agarose gel electrophoresis (Bio-Rad Laboratories, Inc, Hercules, CA, United States). Additionally, the extracted DNA concentration was assessed on a Qubit (Life Technologies, Grand Island, NY, United States) using the High Sensitivity DNA Kit (Agilent Technologies, Santa Clara, CA, United States). The extracted DNA was then subjected to Fluidigm Access Array Amplification (Fluidigm Corporation, South San Francisco, CA, United States). The primer sequences F357 -for (CCTACGGGNGGCWGCAG) and R805-rev (GACTACHVGGGTATCTAATCC) were designed with an attached eight base barcode sequence that was unique to each sample to amplify the V3-V4 hypervariable region of the 16S rRNA gene. Additionally, a total of seven primer sets targeting seven different ARGs conferring resistance to the most popular antimicrobial classes used in the swine industry, were used (Supplementary Table S1). Additionally, the primer sequences F357 -for (CCTACGGGNGGCWGCAG) and R805-rev (GACTACHVGGGTATCTAATCC) were designed with an attached eight base barcode sequence that was unique to each sample to amplify the V3-V4 hypervariable region of the 16S rRNA gene. The mastermix for PCR amplification was prepared using the Roche High Fidelity Fast Start Kit and 20x Access Array loading reagent according to Fluidigm protocols. PCR reactions consisted of DNA sample, 20X Access Array Loading Reagent, forward and reverse primer, Fluidigm Illumina linkers with unique barcode, and water to a final volume of 100 μl. PCR reactions were performed on a Fluidigm Biomark HD^TM^ PCR machine (Fluidigm Corporation, South San Francisco, CA, United States; Supplementary Table S2). Amplicons were purified on a Fragment Analyzer (Advanced Analytics, Ames, IA, United States) to confirm amplicon size. The final fluidigm pools were quantitated by qPCR on a BioRad CFX Connect Real-Time System (Bio-Rad Laboratories, Inc., Hercules, CA, United States). Samples were then pooled in equimolar ratio, spiked with 15% non-indexed PhiX control library, and loaded onto the MiSeq V3 flowcell at a concentration of 8 pM for cluster formation and sequencing. The final genomic libraries were then sequenced from both ends following manufacturer’s guidelines (Illumina, Inc., San Diego, CA, United States) at the DNA Services lab at the W. M. Keck Center for Comparative and Functional Genomics (University of Illinois at Urbana–Champaign, Urbana, IL, United States).

### Sequence Data Processing and Microbial Community Analysis

The raw sequence data were preprocessed from Illumina base call (bcl) files into compressed paired-end read fastq files (2 × 300) using bcl2fastq 1.8.4 (Illumina, San Diego, CA, United States) without demultiplexing, and then sorted by initial PCR-specific primer using a custom in-house pipeline. The generated bcl files were converted into demultiplexed compressed fastq files using bcl2fastq 1.8.4 (Illumina, San Diego, CA, United States). A secondary pipeline decompressed the fastq files, generated plots with quality scores using FastX Tool Kit^1^. Trimmomatic (v. 0.38) was used to trim the low-quality base at the overlapping end of the raw sequence reads (Bolger et al., 2014). Barcode and sequencing primers were also trimmed from the raw sequence reads. After preprocessing, the 16S rRNA gene sequences were analyzed using Quantitative Insights into Microbial Ecology (QIIME v.1.9.1) software^2^ (Caporaso et al., 2010). Raw sequence reads were quality filtered using the following quality criteria; minimum sequence length equal 200, maximum sequence length equal 1000, a Phred score of less than 25, maximum number of ambiguous bases equal 6 and homopolymer runs of >6 bp (Bokulich et al., 2012). The open-reference operational taxonomic unit (OTU) clustering was conducted in QIIME at 97% similarity using UCLUST clustering (v1.2.22q) (Edgar, 2010), and taxanomy was assigned using the Silva reference database (v.132) (Quast et al., 2013). Chimeric sequences were detected and removed using UCHIME (v. 6.1) prior to downstream analysis (Edgar et al., 2011). One sample from the TUL-treated piglets was not included in the analysis due to unsuccessful sequencing. Two OTUs detected as a contaminant in negative controls (classified as *Stenotrophomonas* and *Xanthomonas*) were removed prior to analyses. The alpha diversity (within community) were calculated within QIIME using the number of OTUs per sample and the Shannon diversity index. To standardize our analysis due to uneven sequencing depth, all samples were randomly subsampled to 1358 sequences per sample. To compare overall microbiota composition among groups, a beta diversity analysis was performed considering the abundance of each detected OTUs in each sample using weighted UniFrac distances and was displayed using principal coordinate analysis (PCoA). Finally, a Venn diagram was generated for graphical descriptions of the number of unique and shared OTUs between treatment groups.

Statistical analysis and graphing were performed using PAST version 3.13, JMP 13 (SAS Institute Inc.) and RStudio (version 1.1.383, R Studio, Inc., Boston, MA, United States). Data were logarithmically transformed or ranked when necessary to achieve normality and homogeneity of variance prior to statistical analyses. Significance difference was stated at *P* < 0.05. Statistical comparisons of weighted UniFrac distances between treatment groups at different sampling days were determined using analysis of similarity (ANOSIM) with 9999 permutations and Bonferroni corrected *P*-values in PAST version 3.13. Due to the fact that the same piglets were sampled repeatedly over the course of the study, repeated measures ANOVA with *post hoc* Tukey’s honestly significant difference (HSD) pairwise comparisons were performed to compare the difference in microbial relative abundance and alpha diversity indices between the treatment groups. To further identify taxa that were significantly different between the different time points in the same groups and between the groups at the same time point, the OTUs abundance were assessed using the linear discriminant analysis (LDA) effect size (LEfSe) pipeline in Galaxy^3^ (Segata et al., 2011). We then compared the overall microbial communities between the treatment groups using stepwise discriminant analysis in JMP 13 (SAS Institute Inc.). For this analysis, the relative abundances of different bacterial genera in each group were used as a covariate, and treatment groups were used as the categorical variable. The discriminant analysis was used to determine how equivalent samples, from animals in different groups, were differentiated from one another, and was illustrated using canonical loading plots.

### Prediction of the Metagenome Functions Profiles

The metagenomic prediction of functional profiles based on 16S rRNA gene composition was done with Phylogenetic Investigation of Communities by Reconstruction of Unobserved States (PICRUSt v1.0.06) (Langille et al., 2013). Closed reference OTUs were taxonomically assigned against the Greengenes (v13.5) database, normalized by copy number, and gene features were predicted at level 2 and level 3 Kyoto Encyclopedia of Genes and Genomes (KEGG) orthology groups (Kotera et al., 2012). The unclassified functional categories were eliminated from the analysis. The difference in overall predictive function gene profiles among groups were compared with Statistical Analysis of Metagenomic Profiles software (STAMP v2.1.3) (Parks et al., 2014). Two-sided Welch’s *t*-test and Benjamini–Hochberg FDR correction were used in two-group analysis and ANOVA with the Tukey–Kramer test and Benjamini–Hochberg correction were chosen for multiple-group analysis. Differences were considered significant at *P* < 0.05. Principal component analysis (PCA) and heatmap diagram were also performed using STAMP and MicrobiomeAnalyst respectively (Dhariwal et al., 2017).

### Selected Antimicrobial Resistance Genes Quantification

For ARGs sequence classification, we have developed a customized version of the Antibiotic Resistance Gene Database (ARG-ANNOT) that incorporated all sequences of the seven ARGs that used in this study. The customized ARG-ANNOT database was used to align the seven ARGs raw sequences reads obtained from the Illumina sequencing according to the used primers. The ARG sequence depth and coverage for each ARG were also counted. To avoid bias, normalization of the ARGs reference sequence length by the 16S rRNA gene sequence length was conducted. The abundance of ARGs was expressed as ARG copy number per 16S rRNA gene copy. The relative abundance of ARGs was calculated using the following equation (Li B. et al., 2015):

Abundance=∑1nNARG−like sequenceX Lreads/LARG reference sequenceN16S sequenceX Lreads/L16S sequence

The difference in ARGs abundance, between treatment groups at different sampling days were analyzed using repeated measures ANOVA with pairwise *post hoc* Tukey’s HSD comparisons in PAST version 3.13. Dunnett’s multiple comparisons procedure was also used to compare the mean ARGs abundance in different treatment groups (CCFA, CHC, OTC, PPG, and TUL) at each sampling day (0, 5, 10, 15, and 20), against the CONT group at the same time point. The difference in overall ARGs abundance among treatment groups were compared using PCA fitted in STAMP software (Parks et al., 2014). Differences with a value of *P* < 0.05 were considered significant.

### Accession Numbers

Raw paired-end Fastq sequence data obtained in this study were submitted to the Sequence Read Archive of the NCBI under bio-project accession number PRJNA407634.

## Results

### Impact of Antimicrobial Treatment on Body Weight Gain and Overall Mortality Ratio

There were no significant differences in the average daily weight gain between the treatment groups (CCFA, CHC, OTC, PPG, and TUL) and CONT over the first 20 days of life (*P* > 0.05, Supplementary Figure S1A). Compared to CONT, the treated piglets showed non-significant changes in the overall mortality ratios (*P* > 0.05, Supplementary Figure S1B). However, TUL-treated piglets showed an increase in the mortality during the time period from 15 to 20 days of life (Supplementary Figure S1B). Our results showed that the early life antimicrobial intervention failed to affect mortality or the average daily weight gain in the neonatal piglets.

### Summary of Sequence Data Analysis

After quality filtering and removal of low-quality sequences, a total of 2,508,268 sequences were obtained from all samples. The number of sequences per sample ranged from 5307 to 48524 (mean ± SD, 15201.624 ± 7324.965). Using 97% similarity, 1296 OTUs were identified among all samples. Collectively, most OTUs were shared among the treatment groups with only 8, 5, 18, 11, 4 and 7 OTUs uniquely identified in piglets from the CONT, TUL, CCFA, CHC, PPG, and OTC group, respectively (Supplementary Figure S2).

### Microbial Taxa Affected by Early Life Antimicrobial Intervention

**FIGURE 1:**
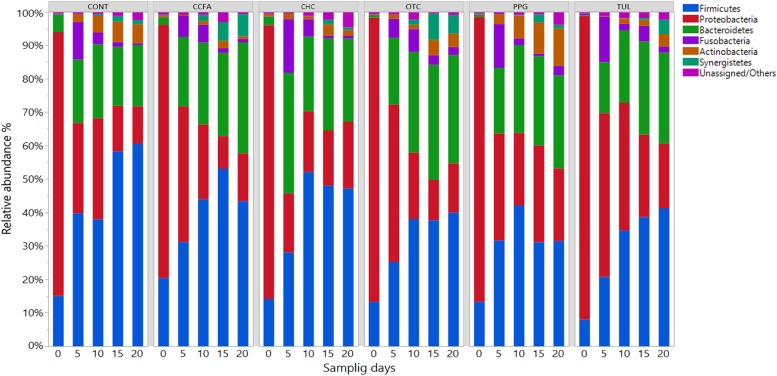
Taxonomic classification of 16S rRNA gene sequences at the phylum level for control (CONT, *n* = 4), ceftiofur crystalline free acid (CCFA, *n* = 4), ceftiofur hydrochloride (CHC, *n* = 4), oxytetracycline (OTC, *n* = 4), procaine penicillin G (PPG, *n* = 4) and tulathromycin (TUL, *n* = 4) treated piglets at each sampling time points. Only those bacterial phyla that averaged more than 1% of the relative abundance across all samples are displayed.

At the phylum level, the microbial composition in all treatment groups varied according to ages (Figure 1). At day 0, Proteobacteria was the most predominant phylum, representing 79, 76, 82, 85, 85, and 91 % of all bacterial populations in CONT, CCFA, CHC, OTC, PPG, and TUL respectively. While at day 20, Firmicutes was the most relatively abundant phylum, representing 61, 43, 47, 40, 32, and 41% of all bacterial populations in CONT, CCFA, CHC, OTC, PPG, and TUL, respectively. Compared to the CONT group, TUL-treated piglets exhibited a lower relative abundance of Actinobacteria at day 5 (*P* = 0.029). Furthermore, CONT piglets had a higher relative abundance of Firmicutes compared to those in PPG group at days 15 and 20 (*P* = 0.031 and 0.016), respectively.

**FIGURE 2:**
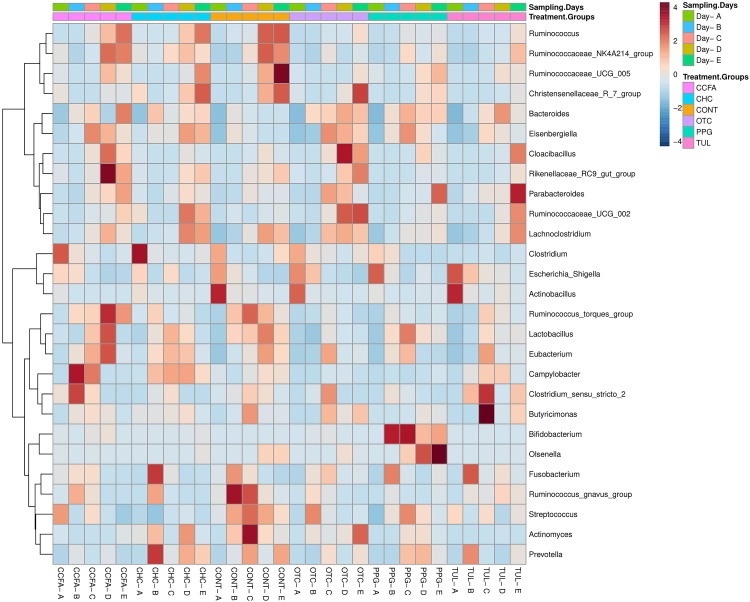
Heatmap cluster analysis of the most relatively abundant genera for control (CONT, *n* = 4), ceftiofur crystalline free acid (CCFA, *n* = 4), ceftiofur hydrochloride (CHC, *n* = 4), oxytetracycline (OTC, *n* = 4), procaine penicillin G (PPG, *n* = 4) and tulathromycin (TUL, *n* = 4) treated piglets at each sampling time point. Only those bacterial genera that averaged more than 1% of the relative abundance across all samples are displayed.

At the genus level, a total of 189 genera were identified from all samples. The core fecal microbial community (defined as the genera found at a relative abundance of > 1% in all treatment groups) at the baseline (day 0) was comprised of common fecal microbial genera including *Escherichia–Shigella* (41.24%), *Clostridium* (17.33%), *Fusobacterium* (4.58%), *Bacteroides* (3.39%), *Actinobacillus* (3.04%), *Streptococcus* (3.01%), and *Lactobacillus* (2.44%). A hierarchically clustered heatmap of the most predominant microbial communities at the genus level is shown in (Figure 2). Compared to CONT, the TUL-treated piglets showed a decline in the relative abundance of *Ruminococcus* at day 15 and *Actinomyces* at days 10 and 20 of life. In contrast, the TUL-treated piglets had an increased proportion of *Escherichia-Shigella* at day 5 and *Bacteroides* at day 14. In CCFA group, the treated piglets had an increased proportion of *Campylobacter* at day 5, *Rikenellaceae RC9 gut group* at day 15 and a reduction in the proportion of *Lactobacillus* at day 5, *Streptococcus* at day 5, *Prevotella* at day 15. In CHC group, the piglets had a lower relative abundance of Streptococcus at day 5 and an increased proportion of *Campylobacter* at day 10. The OTC-treated piglets exhibited an increase in the relative abundance of *Escherichia-Shigella* at day 5, *Bacteroides* at day 15, and a reduction in the relative abundance of *Lactobacillus* at day 5. In PPG group, the piglets showed a reduction in the proportion of *Fusobacterium* at day 10 and *Clostridium* at day 20. The PPG-treated piglets had an increased proportion of *Olsenella* at day 15 and day 20, *Escherichia-Shigella* at day 15, and *Bacteroides* at day 15 and day 20.

**FIGURE 3:**
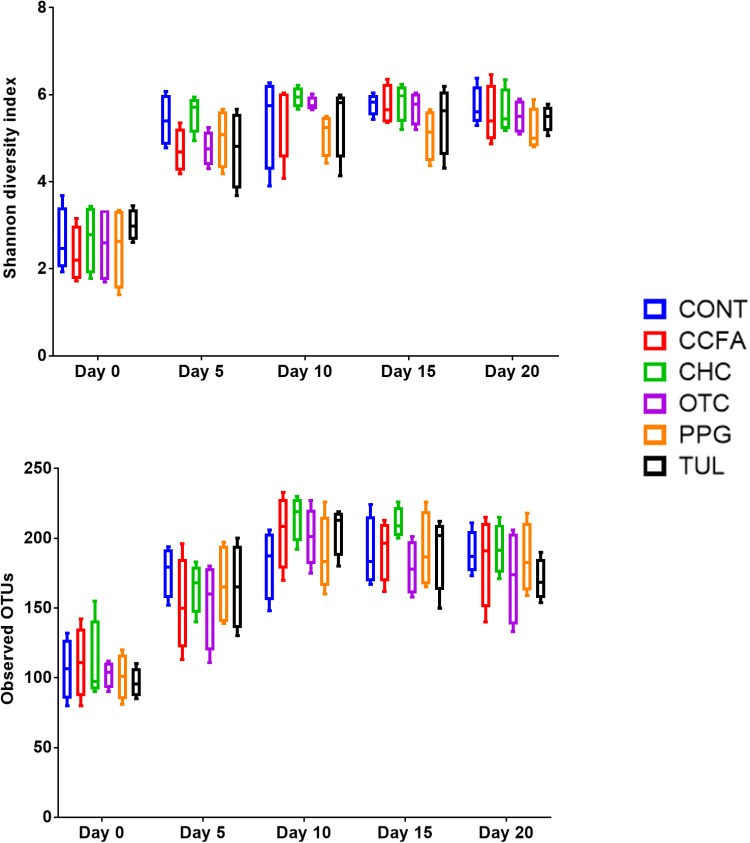
Bacterial diversity indices by treatment groups control (CONT, *n* = 4), ceftiofur crystalline free acid (CCFA, *n* = 4), ceftiofur hydrochloride (CHC, *n* = 4), oxytetracycline (OTC, *n* = 4), procaine penicillin G (PPG, *n* = 4), and tulathromycin (TUL, *n* = 4) at different time points (days 0, 5, 10, 15, and 20).

**FIGURE 4:**
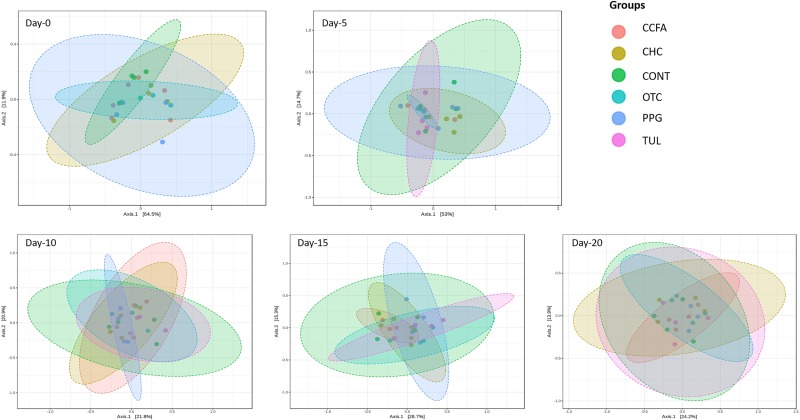
Principal coordinate analysis (PCoA) plot of the weighted Unifrac distances by treatment groups [control (CONT, *n* = 4), ceftiofur crystalline free acid (CCFA, *n* = 4), ceftiofur hydrochloride (CHC, *n* = 4), oxytetracycline (OTC, *n* = 4), procaine penicillin G (PPG, *n* = 4) and tulathromycin (TUL, *n* = 4)] at different sampling days. The percent of variation explained by each coordinate is indicated on the axes. Significance between groups was analyzed using analysis of similarity (ANOISM) with 9999 permutations and Bonferroni corrected *P*-values.

Alpha-diversity was computed using the number of OTUs per sample and the Shannon diversity indices (Figure 3). Collectively, the microbial diversity indices increased with age (*P* < 0.001). Alpha diversity metrics showed non-significant changes between the CONT and treatment groups (Figure 3). Beta diversity analysis showed that the overall fecal microbiota structure at baseline (day 0) did not differ by treatment group (ANOSIM, *P* = 0.17; Figure 4). The early life antimicrobial-induced changes in the microbial community composition were not sufficient to cluster samples by treatment at the different time points as shown by PCoA (ANOSIM, *P* > 0.05; Figure 4). However, there was a significant effect of sampling time on the overall microbial community composition (*P* < 0.0001, *R*^2^ = 0.36) (Supplementary Figure S3).

To further evaluate the potential changes in fecal microbiota associated with early life antimicrobial administration and to determine indicator taxa in each group, differences in the relative abundance of taxa between CONT and treated piglets were compared using LEfSe. Compared to CONT group, 15, 6, 14, 8, and 9 OTUs were identified as indicator taxa in CHC, OTC, TUL, PPG, and CCFA treated piglets respectively (Supplementary Figure S4). Additionally, a number of potential indicator taxa that were differentially represented in the treatment groups at the same age with their LDA scores are depicted in (Supplementary Figure S5). Collectively, the changes in the fecal microbiota structure caused by perinatal antimicrobials intervention are limited to a particular group of microbial taxa.

### Relationships Among the Overall Microbiota Composition of the Six Treatment Groups

**FIGURE 5:**
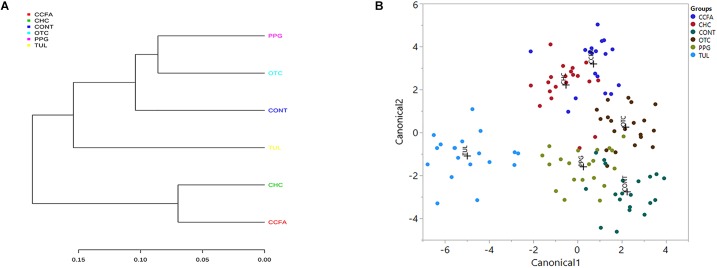
**(A)** Multiple group similarities tree was constructed using weighted Unifrac distances metrics to identify the similarities and differences among antimicrobial treatment groups. **(B)** Discriminant analysis of the overall fecal microbiota composition in different treatment groups [control (CONT, *n* = 4), ceftiofur crystalline free acid (CCFA, *n* = 4), ceftiofur hydrochloride (CHC, *n* = 4), oxytetracycline (OTC, *n* = 4), procaine penicillin G (PPG, *n* = 4) and tulathromycin (TUL, *n* = 4)] across all the time points. Different mean relative abundances of bacterial genera in fecal microbiota were used as covariates, and sampling groups were used as categorical variables. Differences in fecal microbial profiles of different treatment groups are illustrated by canonical 1 and 2.

A multiple group similarities tree was constructed using the Unifrac distance metrics to identify the similarities and differences among the antimicrobial treatment (Figure 5A). Collectively, comparison of the microbiota composition of different treatments group (CCFA, CHC, OTC, PPG, and TUL) showed no significant changes when compared to CONT group (ANOSIM, *P* > 0.05, Supplementary Table S3). However, the taxonomic composition of TUL-treated piglets was separated from the compositions of the CHC and CCFA treated piglets (ANOSIM, *P* = 0.024 and 0.015) respectively (Figure 5A,B). The microbial community structure of the PPG-treated piglets was closest to OTC-treated piglets, indicating a closely community structure between these two treatments (Figure 5A,B). Similarly, samples from the CCFA and CHC piglets were also clustered together indicating that these two treatments resulted in similar community structures (Figure 5A,B).

### Effect of Early Life Antimicrobial on Predicted Microbial Functional Profiles

**FIGURE 6:**
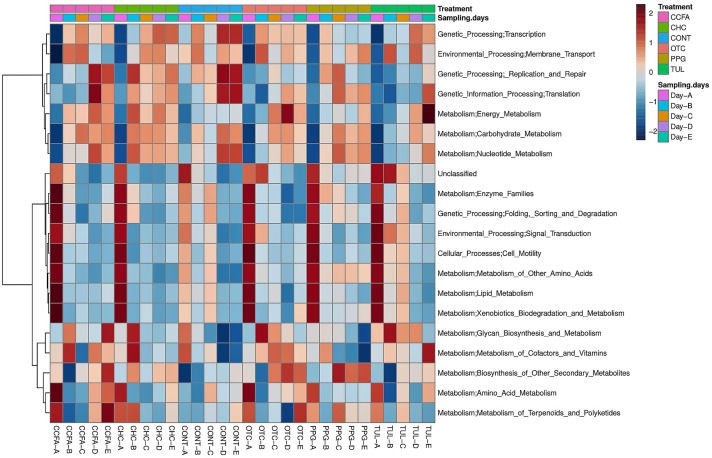
Heatmap cluster analysis of predicted functional pathways (level 2 KEGG) based on differentially abundant functional features between different treatment groups, and at different sampling days.

Predicted functional profiles of the fecal microbial communities in the six groups (CONT, CCFA, CHC, OTC, PPG, and TUL) at the level 2 KEGG pathway were investigated using PICRUSt (Figure 6). Altogether, the PCA plot revealed that the predicted functional genes in each sample varied significantly with age (ANOSIM, *P* < 0.0001, Supplementary Figure S6A). Only, the TUL and CONT groups showed significant differences in the overall predicted KEGG pathways (level 2), particularly in carbohydrate metabolism, glycan biosynthesis, and metabolism and nucleotide metabolism (Supplementary Table S4). Furthermore, PCA of the predicted KEGG pathways (level 2) revealed that samples from CONT and TUL groups were clustered into two distinct groups (ANOSIM; *P* = 0.017; Supplementary Figure S6B). The overall predicted KEGG pathways (level 2) of CCFA, CHC, OTC, and PPG treated piglets showed no significance differences when compared to CONT (ANOSIM; *P* > 0.05, Supplementary Figure S7). Detailed PICRUSt results of the functional gene profiles at KEGG level 3 are depicted in (Supplementary Table S5).

### Effect of Early Life Antimicrobial Administration on Selected ARGs

**FIGURE 7:**
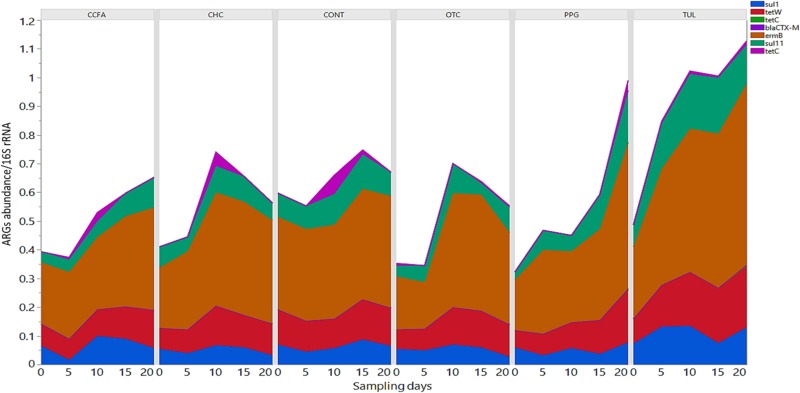
Stacked bar chart showing the relative abundance of antimicrobial resistance genes (*ermB, sulI, sulII, tetC, tetO, bla*_CTX–M_, and *tetW*) of each treatment group at each sampling day.

**FIGURE 8:**
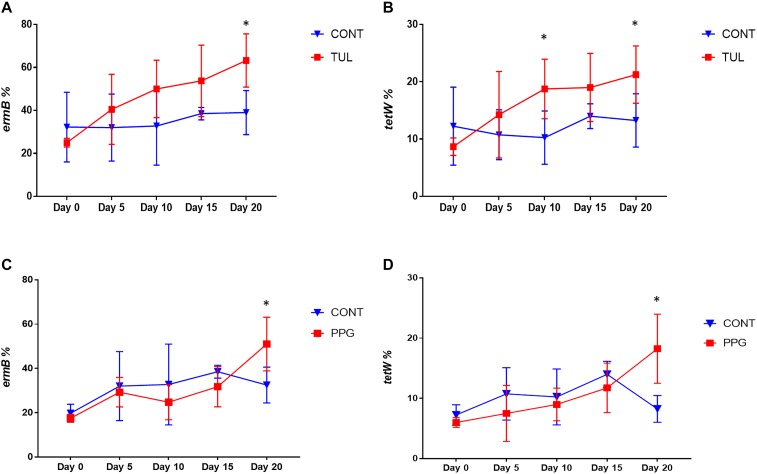
**(A,B)** Line graphs illustrating the difference in relative abundance of *ermB* and *tetW* between the control (CONT, *n* = 4) group, and tulathromycin (TUL, *n* = 4) group at different sampling days. **(C,D)** Line graphs illustrating the difference in abundance of antimicrobial resistance genes (*ermB* and *tetW*) between the control (CONT, *n* = 4) group, and procaine penicillin G (PPG, *n* = 4) treated piglets at each sampling day. ^∗^*P* < 0.05.

In this study, we quantified seven ARGs in relation to the bacterial 16S rRNA gene. All ARGs were detected with the exception of the *bla*_CTX–M_, which was below the limit of quantification in all samples. Across all samples, the most relatively abundant ARGs were *ermB* (33.85%), *tetW* (11.65%), and *Sul*II (9.06%) (Figure 7). Compared to CONT, the early life TUL intervention resulted in a significant increase in the abundance of *tetW* at days 10 and 20 (*P* < 0.05), and *ermB* at day 20 (*P* < 0.05) (Figure 8A,B). PPG-treated piglets exhibited a significant increase in the relative abundance of *ermB* and *tetW* at day 20 of life (*P* < 0.05) (Figure 8C,D). In CCFA, CHC, and OTC groups, comparisons of ARGs abundance showed no significant differences after antimicrobial administration compared to CONT group at the same time point (*P* > 0.05). PCA of the overall ARGs relative abundance (*ermB, tetO, tetW, tetC, sulI*, and *sulII*) revealed that samples from CONT and TUL groups clustered into two distinct groups (ANOSIM; *P* = 0.015, Supplementary Figure S8). The overall relative abundance of ARGs in the CCFA, CHC, OTC, and PPG treated piglets showed no significance difference when compared to CONT group (ANOSIM; *P* > 0.05, Supplementary Figure S8).

## Discussion

The extensive use of antimicrobials has led to emergence of antimicrobial-resistant bacteria and ARGs in the environment, which is thought to pose an imminent threat to animal and human health (Berendonk et al., 2015). Several research studies have also revealed microbial shifts in the swine gastrointestinal microbiota after antimicrobial administration (Kim et al., 2012; Zeineldin et al., 2018a; Zhao et al., 2018). In order to overcome these problems, production systems must adapt to reduce the use of antimicrobials. A key step in reducing antimicrobial use is understanding the mechanism and magnitude by which antimicrobial administration affects the microbial ecosystem, emergence of ARGs, and overall host health. Multiple lines of evidence indicate that the gastrointestinal tract of swine has a complex and diverse microbial ecosystem, where extensive communication between host, mucosal communities, and surrounding environment, occur (Li K. et al., 2017; Zeineldin et al., 2017a). It is therefore crucial to understand how common management practices, including early life antimicrobial administration, may influence this complex ecosystem in animals raised in intensive production systems.

Antimicrobials are used parentally in swine production to control and prevent infectious disease (Pyörälä et al., 2014). Sound scientific evidence shows that antimicrobial intervention can have both detrimental and beneficial effects on host health (Phillips et al., 2004), but this has not been widely studied in neonates. This study used 16S rRNA gene sequencing to quantify the impacts of a single dose of early life antimicrobial on the fecal microbiota structure, and relative abundance of selected ARGs (*ermB*, *tetW*, *tetO*, *tetC*, *sulI*, *sulII*, and *bla*_CTX–M_) in neonatal piglets. In line with other studies, our results demonstrated that the fecal microbial communities in all treatment groups were dominated by Firmicutes, Proteobacteria, and Bacteroidetes at the phylum level (Maradiaga et al., 2018), and by *Escherichia-Shigella*, *Bacteroides*, *Lactobacillus*, *Clostridium*, and *Streptococcus* at the genus level (Maradiaga et al., 2018). In terms of temporal changes, time-dependent dynamics of the piglet’s fecal microbiota were observed. The neonatal piglets at day 0 had a significantly greater proportion of *Escherichia-Shigella, Fusobacterium, Clostridium*, and *Actinobacillus*. The piglets fecal microbiota composition observed in this study at day 0 after birth was similar to that published by Kubasova et al. (2017). Although *Escherichia* and *Clostridium* are often the first genera to colonize the gastrointestinal tract in different animal species (Rodríguez et al., 2015; Slifierz et al., 2015), the presence of *Fusobacterium* in the gut microbiota of 0 day old piglets is of concern since some *Fusobacterium* spp. have been linked to swine dysentery (Durmic et al., 1998). In 20-day-old piglets, *Lactobacillus, Bacteroides, Ruminococcus*, and *Ruminococcaceae* UCG-005 were the most relatively abundant genera, which is similar to previous reports (Slifierz et al., 2015; Kubasova et al., 2017).

Contrary to the disruption of the swine gut microbiota that can result from in feed antimicrobial exposure (Kim et al., 2012, 2016; Looft et al., 2012; Zhao et al., 2018), The observed changes in the developmental dynamics of the fecal microbiota showed antimicrobial-specific variations in both duration and extent. Our findings are generally in line with a previous study that evaluated the impact of antimicrobial treatment on the microbiota composition and resistance gene reservoir (Choo et al., 2018). Using 16S rRNA gene sequencing, Choo et al. (2018) domenstared that the disruption in the oropharyngeal microbiota composition of humans was restricted to a relatively small group of *Actinomyces* species (Choo et al., 2018). In the present study, the reduction in the relative abundance of *Actinomyces* population in response to TUL treatment are in agreement with other *in vitro* studies (Smith et al., 2005). *Actinomyces* spp. are Gram-positive facultative anaerobes that consume lactate and frequently reside in the female genital tract, and gastrointestinal tract of healthy individuals (Takahashi and Yamada, 1999; Smith et al., 2005).

Of particular interest in our results is the decrease in the proportion of *Ruminococcus* in the CCFA, CHC and TUL-treated piglets compared with CONT group. Members of *Ruminococcus* genera are commonly associated with gut health, through generation of short-chain fatty acid that play an important role in reduction of colonization of many opportunistic pathogens (Yu et al., 2018). Additionally, TUL, PPG and OTC treated piglets showed an increased abundance of *Escherichia-Shigella* and *Bacteroides*. *Escherichia* spp. are commonly found in farm environment and are considered indigenous to the piglets gut microbiota (Yang et al., 2004). *Escherichia* spp. can be pathogenic, and include many species associated with neonatal and post weaning diarrhea in swine (Bischoff et al., 2002; Chapman et al., 2006). Similarly, an increase in the abundance of *Bacteriodes* spp. during early life is considered disease predisposing condition (Korpela et al., 2016; Tran et al., 2018). Further studies evaluating the role of *Bacteriodes* and *Escherichia*, either as markers of gastrointestinal dysbiosis after antimicrobial treatment, are warranted. The PPG-treated piglets also exhibited an increase in the proportion of *Olsenella* at day 15 and day 20. The *Olsenella* genus was first proposed by (Dewhirst et al., 2001), and has recently been reclassified to the *Atopobiaceae* family within the *Coriobacteriales* order and *Coriobacteriia* class (Gupta et al., 2013). Members of the *Olsenella* genus are Gram positive rods that produce skatole, a compound responsible for boar taint and off-flavor taint, which released upon heating meat from male pigs (Li X. et al., 2015). In the CCFA and CHC groups, piglets had an increased proportion of *Campylobacter* at day 5 and day 10 respectively. *Campylobacter* spp., are considered one of the common causes of human enteritis (Dicksved et al., 2014) and swine dysentery (Yang et al., 2017). Taken together, our result suggests that early life antimicrobial intervention may make the gastrointestinal tract more susceptible to potential pathogenic bacteria. While it is difficult to understand whether the short-term moderate changes in the developmental dynamics of gastrointestinal microbiota observed in this study have any significant long-term impacts on the health and production of the growing piglets, the significance of antimicrobial-induced microbial shift have been well documented by other researchers (Schokker et al., 2015).

Bacterial diversity is often used as a crucial measure of functional resilience and homeostasis of gastrointestinal microbial ecosystem (Lozupone et al., 2012). Bacterial diversity indices suggest that the piglet fecal microbiota was rich and diverse and underwent intricate development during the first 20 days of life. Similarly, the gastrointestinal tract of piglets during early life, showed an age-dependent manner of microbial population evolution and acquisition (Bian et al., 2016). In line with other studies, our result showed that there was no significant changes in the overall microbial community composition between treatment groups at each time point as measured by beta diversity analysis (Zhang et al., 2016; Li P. et al., 2017). In contrast, Looft et al. (2014) observed a significant changes in diversity indices after early life carbadox administration of in 6-weeks-old piglets. The discrepancies between the present study and previous research might have resulted from the use of different type of antimicrobial, dosage, route of administration, and different environmental conditions (Li P. et al., 2017).

To identify indicator taxa that are significantly discriminated between CONT and other treatment groups, we used a well-established approach, LEfSe, to identify bacterial taxa of interest for further analysis (Segata et al., 2011). In this study, LEfSe revealed 15, 6, 14, 8, and 9 OTUs as indicator taxa in CHC, OTC, TUL, PPG, and CCFA treated piglets respectively. These results further support the concept that the shifts in the fecal microbiota structure caused by perinatal antimicrobial intervention are modest and are limited to a particular group of microbial taxa. We also used PICRUSt to predict the fecal metagenome and identified potential functional pathways that were significantly different between treatment groups. Similar to highly diverse and developed fecal microbiota, predicted functional pathways differed by time point. While these are only presumptions, based on the predicted functional features of the taxonomically assigned microbial population in our study, similar shift have been noted after different antimicrobial therapy in humans (Pérez-Cobas et al., 2013). The significant enrichment in some functional pathways after different antimicrobial administration implied that these functional features might play a crucial role under stress conditions (Wang and Quinn, 2010). Similarly, Perez-Cobas et al. (2012) demonstrated an increase in functional genes belonging to carbohydrate metabolism and energy metabolism/sugars category during antimicrobial treatment (Perez-Cobas et al., 2012). Further investigations into the functional profiles associated with microbial community changes (i.e., which community members have the same functional features and could alternate for one another), either by shotgun metagenomics, direct metabolites measurement or by transcriptome analysis, will be an essential next step to better understand the effect of early life antimicrobial interventions on microbiota function in piglets.

In this study, we assessed carriage of seven different ARGs genes (*tetW, tetO, tetC, sulI, ermB, sulII*, and *bla*_CTX–M_) in relation to the bacterial 16S rRNA gene, based on their identification in previous research (Supplementary Table S1). The tested ARGs belongs to the most abundant type of these ARGs confer resistance to macrolides, beta lactams, sulfonamide and tetracycline, and can be carried by common members of the gut microbiota (Li et al., 2016). Our results demonstrate that the ARGs were present in the piglet’s gut microbiota from the first day of life. Compared to CONT group, the TUL and PPG treated piglets exhibited a significant increase in the relative abundance of the *ermB* gene. This finding is in line with the increased carriage of *ermB* after long-term administration of erythromycin in healthy individuals (Choo et al., 2018). The *ermB* gene can be horizontally transferred between the commensal microbiota via transformation or conjugation, permitting commensal microbiota to serve as a resistance reservoirs (Roberts et al., 2011). Additionally, the TUL and PPG treated piglets exhibited a significant increase in the relative abundance of *tetW*, which encodes for a ribosomal protection protein. Interestingly, the change in the proportion of *ermB* and *tetW* had a similar temporal pattern. This might indicate that these genes are linked together on the same mobile genetic element (Rubio-Cosials et al., 2018). These findings suggest that single dosages of TUL and PPG can increase the relative abundance of ARGs conferring resistance to antimicrobials that are not administrated. Moreover, increases in the levels of transmissible ARGs within the developing fecal microbiota highlight the potential of the gut to act as a resistance reservoir (Looft et al., 2012).

Our study had a number of experimental limitations that should be considered. The sequencing analysis was conducted on a relatively small number of piglets, though similar to other published sequencing studies (Yu et al., 2018). Furthermore, our analysis focused on short-term impacts of antimicrobial administration on the fecal microbiota. It would have been interesting to continue to sample the piglets for a longer period after weaning to define how these changes impact future health and productivity of growing piglets. Finally, our study focused on identification of selected ARGs, and we did not evaluate change in the resistome using non-targeted sequencing. Despite these experimental limitations, our study results provide preliminary insight into an area of investigation that could be of great relevance to the swine gut health. Understanding the factors that influence the developmental dynamics of gut microbiota is important for establishing which management approaches could be used to promote and maintain a stable microbial ecosystem during this important phase of production.

## Conclusion

This study demonstrated that antimicrobial intervention had relatively minor effects on the gut microbiota development during early life in comparison to control piglets but alterations were noticeable in particular taxa. However, early life TUL and PPG intervention could promote selection of ARGs in herds. This knowledge may help us to understand the impacts of early antimicrobial exposure on gut microbial composition and development of ARGs in swine management system. Understanding when and how and the gut microbiota changes in response to antimicrobial administration will aid in the development of new antimicrobial alternatives.

## Author Contributions

JL and BA designed the experiment. BrB, AM, BeB, MZ, and JL conducted the experiment. MZ, AM, BrB, and BeB carried out the laboratory analyses. MZ and AM conducted the data analysis. MZ wrote the manuscript. All authors edited and approved the manuscript for submission.

## Conflict of Interest Statement

The authors declare that the research was conducted in the absence of any commercial or financial relationships that could be construed as a potential conflict of interest.

## Footnotes

1 http://hannonlab.cshl.edu/fastx_toolkit/

2 http://qiime.org/

3 https://huttenhower.sph.harvard.edu/galaxy/

## Supplementary Material

FIGURE S1 **(A)** Bar graph illustrating body weight (kg) at day 0 and day 20, and average weight gain from day 0 to day 20 in different treatment groups. **(B)** Bar graph illustrating the mortality percent of piglets from day 0 to day 5 (day 0–5), from day 5 to day 10 (day 5–10), from day 10 to day 15 (day 10–15), and from day 15 to day 20 (day 15–20) in different treatment groups. There was no significant change in the average daily weight gain and overall mortality ratio (*P* > 0.05).

FIGURE S2 Venn diagram depicting the common and unique OTUs among the different treatment groups (CONT, CCFA, CHC, OTC, PPG, and TUL). A total of 842 OTUs were represented core microbiota and shared between all treatment groups.

FIGURE S3 Principal coordinate analysis (PCoA) for different sampling days (0, 5, 10, 15, and 20). The percent variation explained by each coordinate is indicated on the axes. The individual data points from which represent total fecal microbiota compositions of each piglet are also depicted. Significance between groups was analyzed using analysis of similarity (ANOISM) with 9999 permutations and Bonferroni corrected *P*-values.

FIGURE S4 LDA Effect Size (LEfSe) analysis of fecal microbiota depicting the top OTUs with the highest linear discriminant analysis LDA score log10 ≥ 2.0 that discriminate between the CONT group and CCFA, CHC, OTC, PPG, and TUL treated piglets. Each color refers to each group and its corresponding indicator taxa.

FIGURE S5 Identification of indicator bacterial taxa associated with statistically significant differential abundance between the different treatment groups (CONT, CCFA, CHC, OTC, PPG, and TUL) at different sampling days. The top OTUs with the highest LDA score log10 ≥ 2.0 that discriminate between the CONT group and CCFA, CHC, OTC, PPG, and TUL treated piglets at each time point are depicted. Each color refers to each group and its corresponding indicator taxa.

FIGURE S6 Principal component analysis for overall functional gene profiles at different sampling days (days 0, 5, 10, 15, and 20). **(B)** PCA for overall functional gene profiles between the CONT and TUL-treated piglets. The percent variation explained by each principal component is indicated on the axes.

FIGURE S7 Principal component analysis for the overall predicted functional gene pathways between CONT and different treatment groups (CCFA, CHC, OTC, and PPG) across all time points.

FIGURE S8 Principal component analysis for overall selected antimicrobial resistance genes (*ermB, sulI, sulII, tetC, tetO*, and *tetW*) between CONT group and CCFA, CHC, OTC, PPG, and TUL treated piglets.

TABLE S1 Primers targeting selected antibiotic resistance genes used in this study.

TABLE S2 Access Array cycling program without imaging (Fluidigm Biomark HD PCR machine) for amplifying the primer/sample combinations.

TABLE S3 The result of nonparametric ANOSIM test (analysis of similarities) with 9999 Monte Carlo permutations to evaluate the UniFrac distances significance between different treatment groups.

TABLE S4 The difference in mean relative abundance of function gene profiles in fecal microbiota at level 2 KEGG pathway between the CONT and TUL groups.

TABLE S5 Summarized PICRUSt results of mean relative abundance of function gene profiles in fecal microbiota at level 3 KEGG pathway in all treatment groups (CONT, CCFA, CHC, OTC, PPG, and TUL).
