# Supplementary figures and images for: Effect of Single Dose of Antimicrobial Administration at Birth on Fecal Microbiota Development and Prevalence of Antimicrobial Resistance Genes in Piglets
Source: Front Microbiol. 2019 Jun 19;10:1414. doi: 10.3389/fmicb.2019.01414 (PMC6593251; doi:10.3389/fmicb.2019.01414)

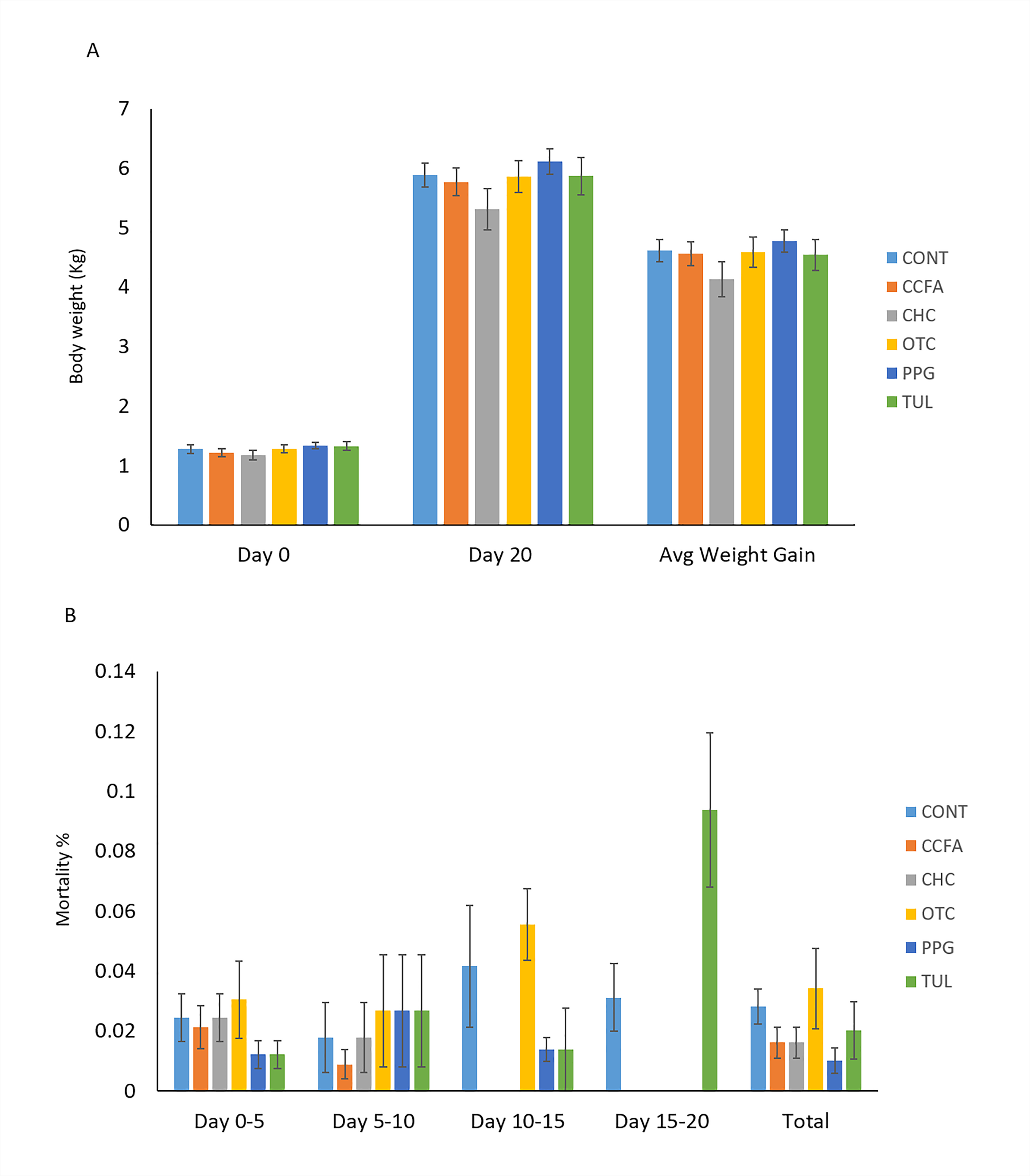

Supplement: FIGURE S1 — (A) Bar graph illustrating body weight (kg) at day 0 and day 20, and average weight gain from day 0 to day 20 in different treatment groups. (B) Bar graph illustrating the mortality percent of piglets from day 0 to day 5 (day 0–5), from day 5 to day 10 (day 5–10), from day 10 to day 15 (day 10–15), and from day 15 to day 20 (day 15–20) in different treatment groups. There was no significant change in the average daily weight gain and overall mortality ratio (P > 0.05). [file Image_1.TIF]

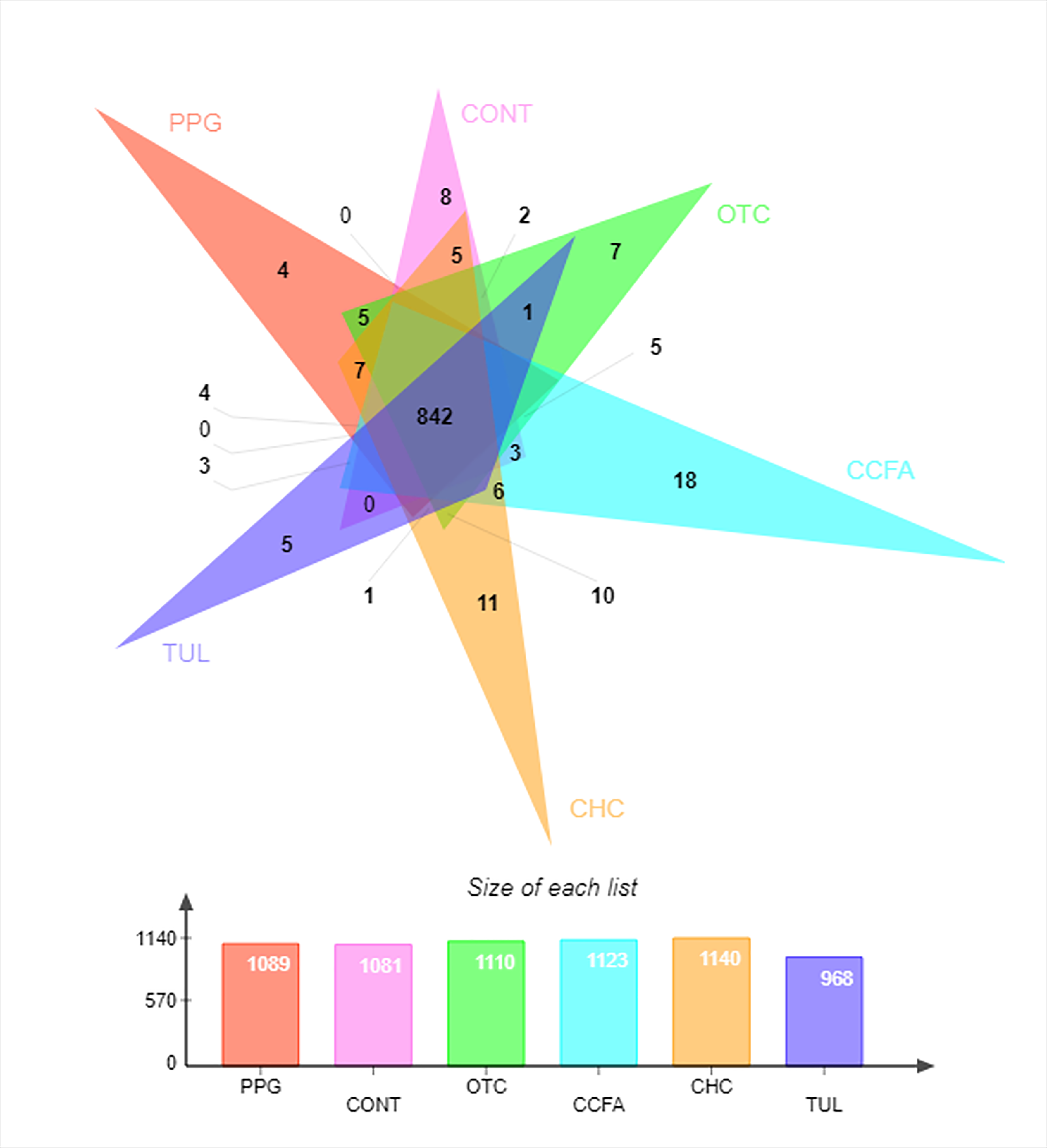

Supplement: FIGURE S2 — Venn diagram depicting the common and unique OTUs among the different treatment groups (CONT, CCFA, CHC, OTC, PPG, and TUL). A total of 842 OTUs were represented core microbiota and shared between all treatment groups. [file Image_2.TIF]

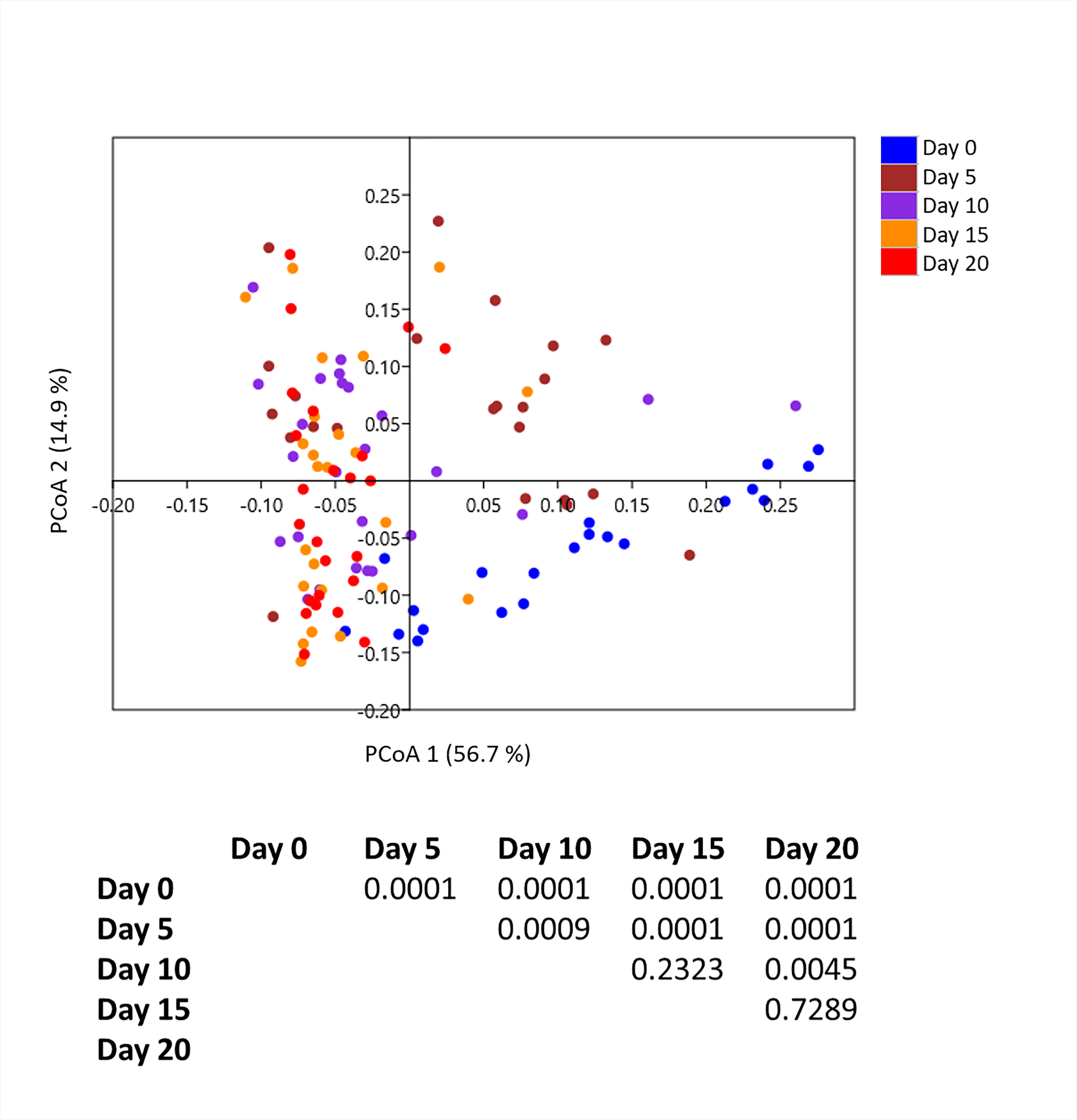

Supplement: FIGURE S3 — Principal coordinate analysis (PCoA) for different sampling days (0, 5, 10, 15, and 20). The percent variation explained by each coordinate is indicated on the axes. The individual data points from which represent total fecal microbiota compositions of each piglet are also depicted. Significance between groups was analyzed using analysis of similarity (ANOISM) with 9999 permutations and Bonferroni corrected P-values. [file Image_3.TIF]

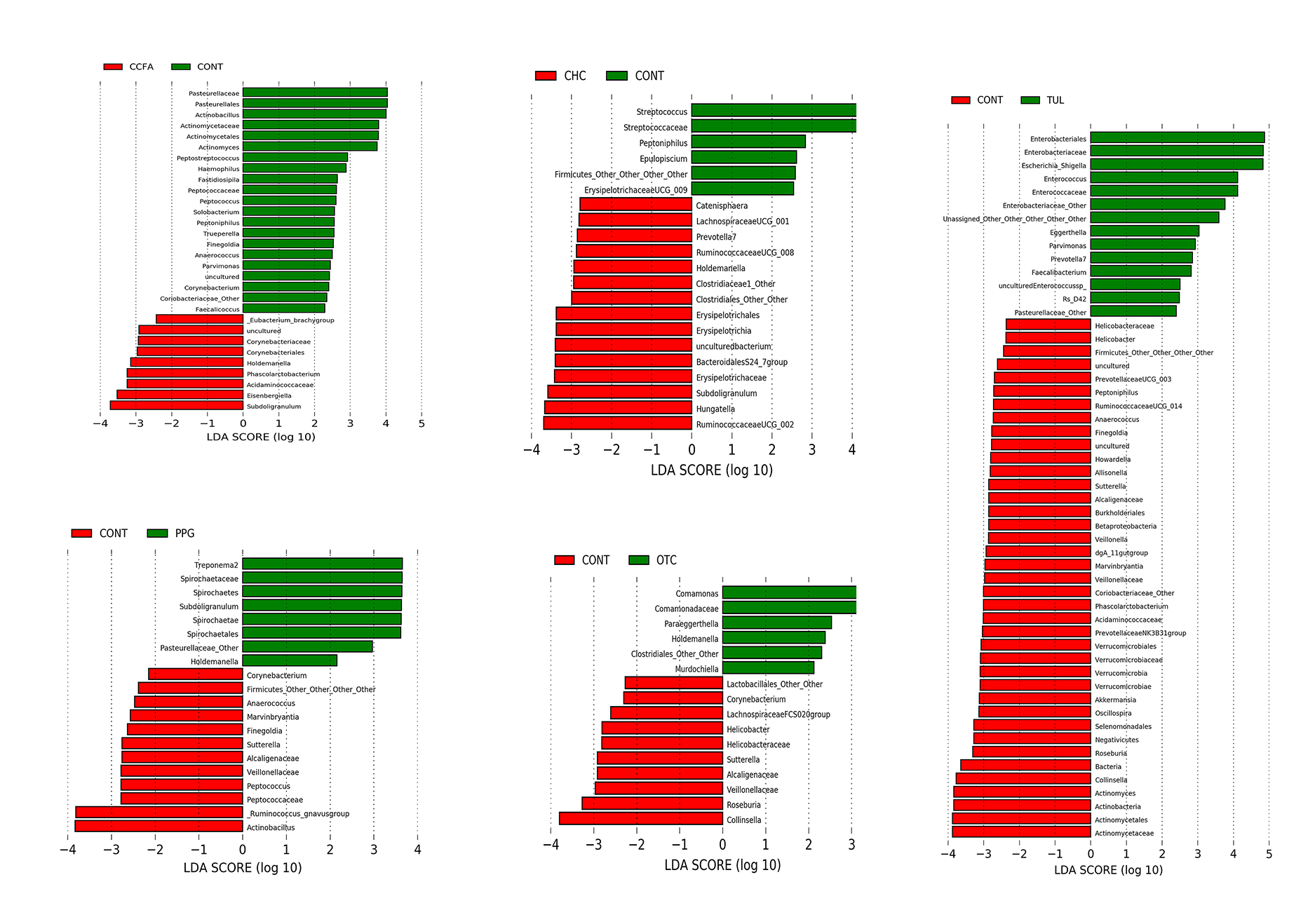

Supplement: FIGURE S4 — LDA Effect Size (LEfSe) analysis of fecal microbiota depicting the top OTUs with the highest linear discriminant analysis LDA score log10 ≥ 2.0 that discriminate between the CONT group and CCFA, CHC, OTC, PPG, and TUL treated piglets. Each color refers to each group and its corresponding indicator taxa. [file Image_4.TIF]

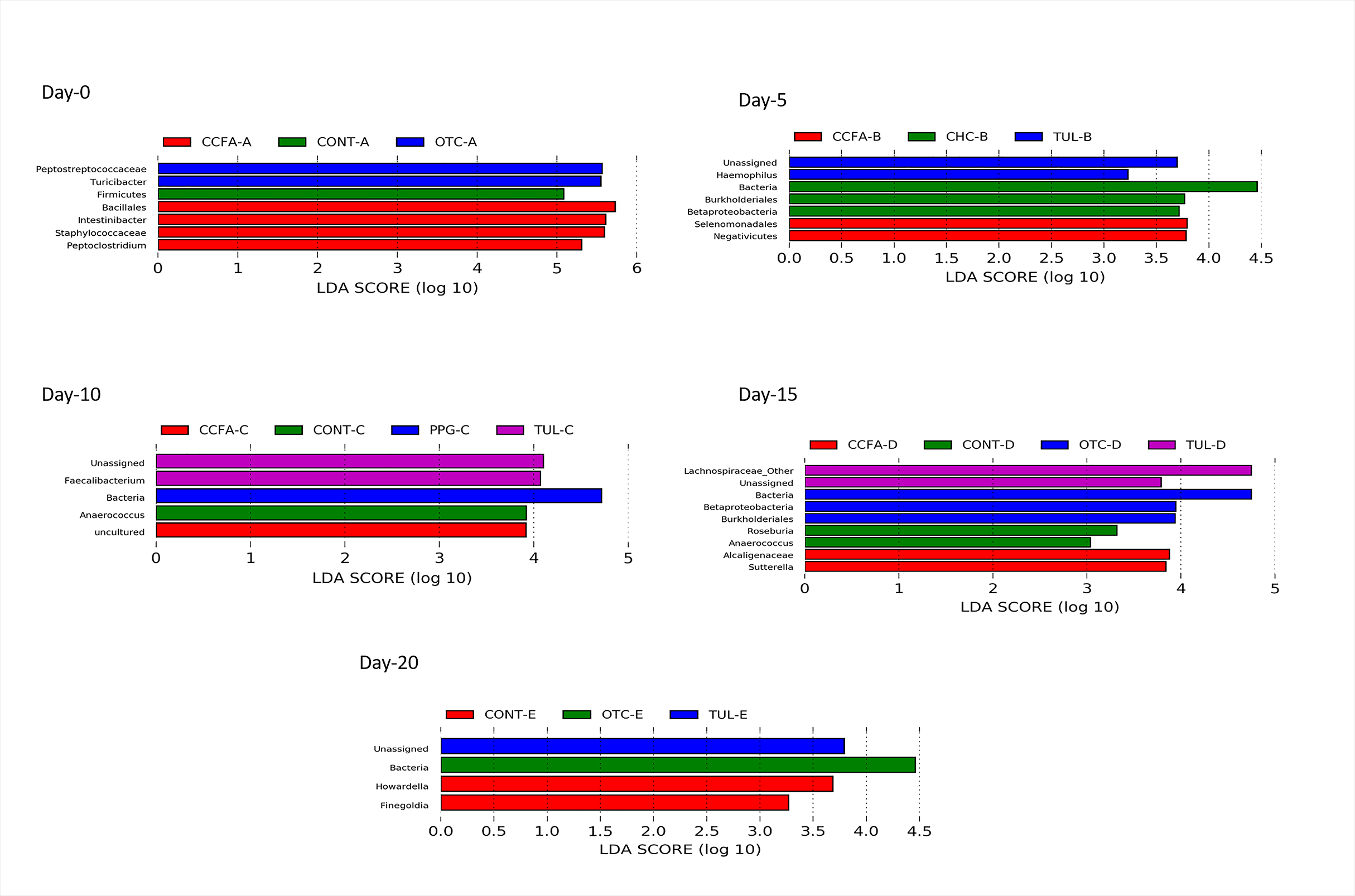

Supplement: FIGURE S5 — Identification of indicator bacterial taxa associated with statistically significant differential abundance between the different treatment groups (CONT, CCFA, CHC, OTC, PPG, and TUL) at different sampling days. The top OTUs with the highest LDA score log10 ≥ 2.0 that discriminate between the CONT group and CCFA, CHC, OTC, PPG, and TUL treated piglets at each time point are depicted. Each color refers to each group and its corresponding indicator taxa. [file Image_5.TIF]

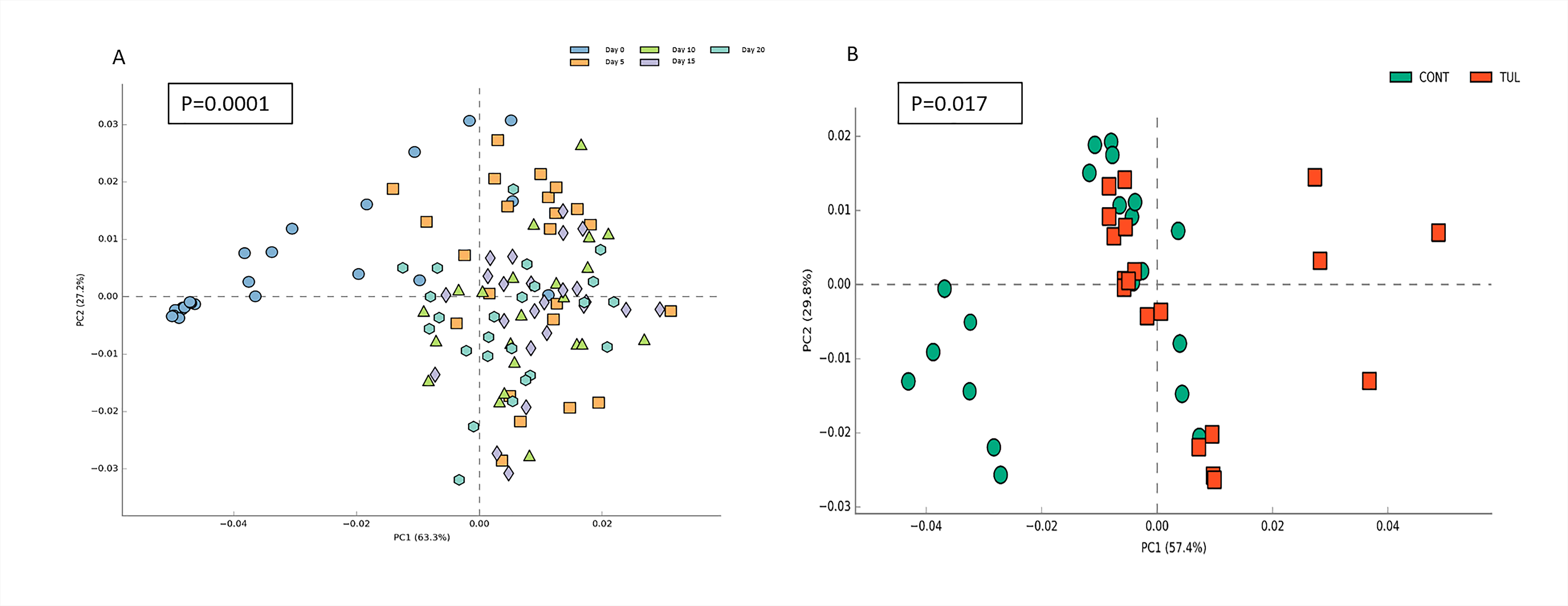

Supplement: FIGURE S6 — Principal component analysis for overall functional gene profiles at different sampling days (days 0, 5, 10, 15, and 20). (B) PCA for overall functional gene profiles between the CONT and TUL-treated piglets. The percent variation explained by each principal component is indicated on the axes. [file Image_6.TIF]

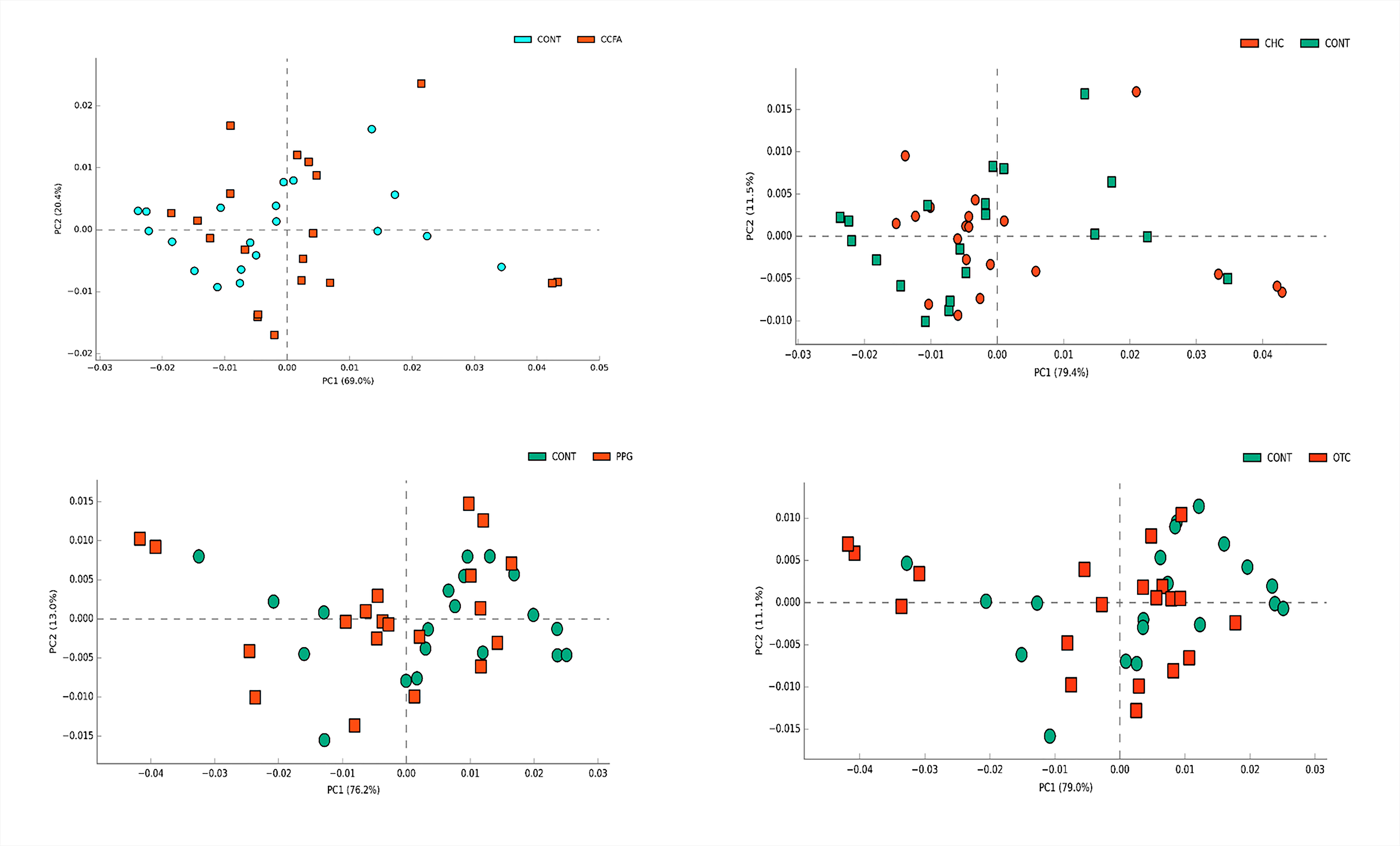

Supplement: FIGURE S7 — Principal component analysis for the overall predicted functional gene pathways between CONT and different treatment groups (CCFA, CHC, OTC, and PPG) across all time points. [file Image_7.TIF]

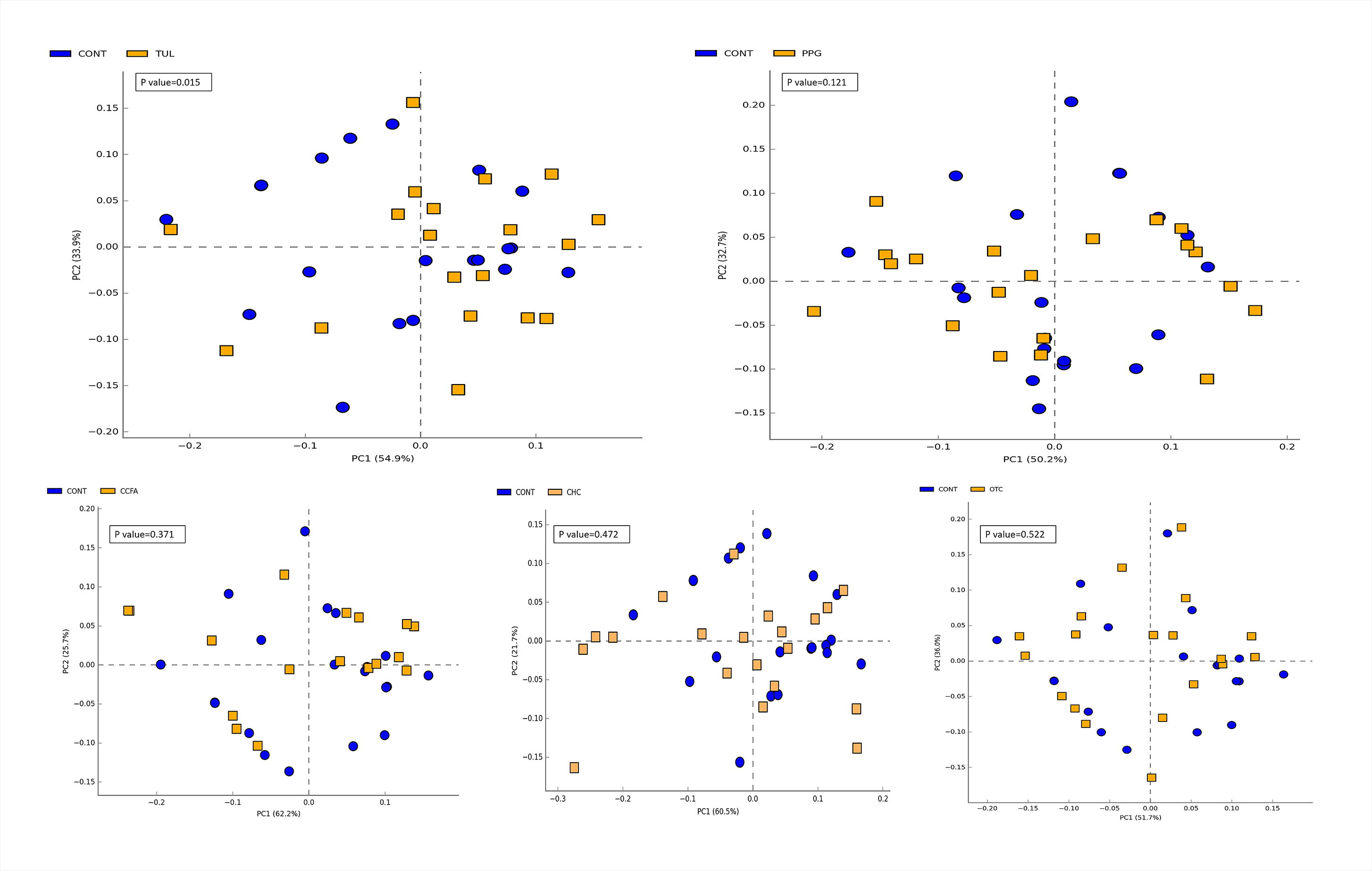

Supplement: FIGURE S8 — Principal component analysis for overall selected antimicrobial resistance genes (ermB, sulI, sulII, tetC, tetO, and tetW) between CONT group and CCFA, CHC, OTC, PPG, and TUL treated piglets. [file Image_8.TIF]
