# Supplementary material for: Effect of Single Dose of Antimicrobial Administration at Birth on Fecal Microbiota Development and Prevalence of Antimicrobial Resistance Genes in Piglets
Source: Front Microbiol. 2019 Jun 19;10:1414. doi: 10.3389/fmicb.2019.01414 (PMC6593251; doi:10.3389/fmicb.2019.01414)
Supplement: TABLE S1 — Primers targeting selected antibiotic resistance genes used in this study. [file Table_1.docx]

**Table S1:** Primers targeting selected antibiotic resistance genes used in this study.

| **Target gene** | **Primer** | **Sequence 5'-3'** | **Amplicon**  **size (bp)** |
| --- | --- | --- | --- |
| tet O | tetO_F | ACGGARAGTTTATTGTATACC | 1920 |
|  | tetO_R | TGGCGTATCTATAATGTTGAC |  |
| tet W | tetW_F | GAGAGCCTGCTATATGCCAGC | 1920 |
|  | tetW_R | GGGCGTATCCACAATGTTAAC |  |
| tet C | tetC_F | GCGGGATATCGTCCATTCCG | 1191 |
|  | tetC_R | GCGTAGAGGATCCACAGGACG |  |
| sul I | sul I_F | CGCACCGGAAACATCGCTGCAC | 840 |
|  | sul I_R | TGAAGTTCCGCCGCAAGGCTCG |  |
| sul II | sul II_F | TCCGGTGGAGGCCGGTATCTGG | 816 |
|  | sul II_R | CGGGAATGCCATCTGCCTTGAG |  |
| erm B | erm B_F | GGTTGCTCTTGCACACTCAAG | 738 |
|  | ermB_R | CAGTTGACGATATTCTCGATTG |  |
| bla _CTX-M_ | CTX-M_F | CTATGGCACCACCAACGATA | 876 |
|  | CTX-M_R | ACGGCTTTCTGCCTTAGGTT |  |
