# Supplementary material for: Effect of Single Dose of Antimicrobial Administration at Birth on Fecal Microbiota Development and Prevalence of Antimicrobial Resistance Genes in Piglets
Source: Front Microbiol. 2019 Jun 19;10:1414. doi: 10.3389/fmicb.2019.01414 (PMC6593251; doi:10.3389/fmicb.2019.01414)
Supplement: TABLE S2 — Access Array cycling program without imaging (Fluidigm Biomark HD PCR machine) for amplifying the primer/sample combinations. [file Table_2.docx]

**Table S2**. Access Array cycling program without imaging (Fluidigm Biomark HD PCR machine) for amplifying the primer/sample combinations.

| **PCR Stages** | **Number of Cycles** |
| --- | --- |
| 50ºC 2 minutes | 1 |
| 70ºC 20 minutes | 1 |
| 95ºC 10 minutes   - 95ºC 15 seconds - 55ºC 30 seconds | 1 |
| 72ºC 1 minute   - 95ºC 15 seconds - 80ºC 30 seconds - 60ºC 30 seconds | 10 |
| 72ºC 1 minute   - 95ºC 15 seconds - 55ºC 30 seconds | 2 |
| 72º 1 minute   - 95ºC 15 seconds - 80ºC 30 seconds - 60ºC 30 seconds | 8 |
| 72ºC 1 minute   - 95ºC 15 seconds - 55ºC 30 seconds | 2 |
| 72ºC 1 minute   - 95ºC 15 seconds - 80ºC 30 seconds - 60ºC 30 seconds | 8 |
| 72ºC 1 minute | 5 |
