# Supplementary material for: Effect of Single Dose of Antimicrobial Administration at Birth on Fecal Microbiota Development and Prevalence of Antimicrobial Resistance Genes in Piglets
Source: Front Microbiol. 2019 Jun 19;10:1414. doi: 10.3389/fmicb.2019.01414 (PMC6593251; doi:10.3389/fmicb.2019.01414)
Supplement: TABLE S3 — The result of nonparametric ANOSIM test (analysis of similarities) with 9999 Monte Carlo permutations to evaluate the UniFrac distances significance between different treatment groups. [file Table_3.DOCX]

**Table S3.** The result of the nonparametric ANOSIM test (analysis of similarities) with 9999 Monte Carlo permutations to evaluate the UniFrac distances significance between the treatment groups.

|  | **CCFA** | **CHC** | **CONT** | **TUL** | **OTC** | **PPG** |
| --- | --- | --- | --- | --- | --- | --- |
| **CCFA** |  | 0.8935 | 0.4816 | **0.0243** | 0.4357 | 0.3249 |
| **CHC** |  |  | 0.3806 | **0.015** | 0.3179 | 0.2128 |
| **CONT** |  |  |  | 0.196 | 0.6121 | 0.1837 |
| **TUL** |  |  |  |  | 0.1124 | 0.0759 |
| **OTC** |  |  |  |  |  | 0.2548 |
| **PPG** |  |  |  |  |  |  |
