# Supplementary material for: Effect of Single Dose of Antimicrobial Administration at Birth on Fecal Microbiota Development and Prevalence of Antimicrobial Resistance Genes in Piglets
Source: Front Microbiol. 2019 Jun 19;10:1414. doi: 10.3389/fmicb.2019.01414 (PMC6593251; doi:10.3389/fmicb.2019.01414)
Supplement: TABLE S4 — The difference in mean relative abundance of function gene profiles in fecal microbiota at level 2 KEGG pathway between the CONT and TUL groups. [file Table_4.DOCX]

**Table S4.** The difference in mean relative abundance of function gene profiles in fecal microbiota at level 2 KEGG pathway between CONT and TUL groups.

| **Functional features at level 2 KEGG pathway** | **CONT** | **TUL** | **P value** |
| --- | --- | --- | --- |
| Amino Acid Metabolism | 19.84% | 19.95% | 0.639 |
| Carbohydrate Metabolism | 22.85% | 20.97% | **0.037** |
| Energy Metabolism | 11.70% | 11.85% | 0.318 |
| Enzyme Families | 4.58% | 4.56% | 0.713 |
| Glycan Biosynthesis and Metabolism | 4.79% | 5.87% | **0.0196** |
| Lipid Metabolism | 6.47% | 6.58% | 0.439 |
| Metabolism of Cofactors and Vitamins | 8.71% | 8.81% | 0.286 |
| Metabolism of Terpenoids and Polyketides | 3.43% | 3.42% | 0.857 |
| Nucleotide Metabolism | 8.47% | 7.02% | **0.0053** |
| Xenobiotics Biodegradation and Metabolism | 4.14% | 4.32% | 0.2995 |
