# Supplementary material for: Effect of Single Dose of Antimicrobial Administration at Birth on Fecal Microbiota Development and Prevalence of Antimicrobial Resistance Genes in Piglets
Source: Front Microbiol. 2019 Jun 19;10:1414. doi: 10.3389/fmicb.2019.01414 (PMC6593251; doi:10.3389/fmicb.2019.01414)
Supplement: TABLE S5 — Summarized PICRUSt results of mean relative abundance of function gene profiles in fecal microbiota at level 3 KEGG pathway in all treatment groups (CONT, CCFA, CHC, OTC, PPG, and TUL). [file Table_5.DOCX]

**Table S5.** Summarized PICRUSt results of mean relative abundance of function gene profiles in fecal microbiota at level 3 KEGG pathway in all treatment group (CONT, CCFA, CHC, OTC, PPG and TUL).

| **Functional features at level 3 KEGG pathway** | **CCFA** | **CHC** | **CONT** | **OTC** | **PPG** | **TUL** |
| --- | --- | --- | --- | --- | --- | --- |
| **Cellular Processes** |  | | | | | |
| Cell Growth and Death;Cell cycle - Caulobacter | 0.45% | 0.45% | 0.47% | 0.44% | 0.45% | 0.43% |
| Cell Growth and Death;Meiosis - yeast | 0.01% | 0.02% | 0.01% | 0.02% | 0.02% | 0.02% |
| Cell Motility;Bacterial chemotaxis | 0.48% | 0.50% | 0.43% | 0.54% | 0.49% | 0.52% |
| Cell Motility;Bacterial motility proteins | 1.29% | 1.33% | 1.13% | 1.49% | 1.42% | 1.53% |
| Cell Motility;Cytoskeleton proteins | 0.30% | 0.31% | 0.32% | 0.28% | 0.28% | 0.26% |
| Cell Motility;Flagellar assembly | 0.52% | 0.53% | 0.44% | 0.58% | 0.55% | 0.61% |
| Transport and Catabolism;Lysosome | 0.11% | 0.11% | 0.09% | 0.12% | 0.10% | 0.11% |
| Transport and Catabolism;Peroxisome | 0.19% | 0.19% | 0.18% | 0.21% | 0.20% | 0.21% |
| **Environmental Information Processing** |  |  |  |  |  |  |
| Membrane Transport;ABC transporters | 3.00% | 2.98% | 3.09% | 3.00% | 2.91% | 2.94% |
| Membrane Transport;Bacterial secretion system | 0.73% | 0.72% | 0.71% | 0.72% | 0.74% | 0.77% |
| Membrane Transport;Phosphotransferase system (PTS) | 0.58% | 0.56% | 0.71% | 0.57% | 0.63% | 0.61% |
| Membrane Transport;Secretion system | 1.65% | 1.61% | 1.57% | 1.70% | 1.75% | 1.84% |
| Membrane Transport;Transporters | 5.99% | 6.04% | 6.30% | 5.90% | 5.85% | 5.83% |
| Signal Transduction;Two-component system | 1.83% | 1.80% | 1.68% | 1.92% | 1.84% | 1.99% |
| Signaling Molecules and Interaction;Bacterial toxins | 0.12% | 0.13% | 0.13% | 0.12% | 0.12% | 0.11% |
| Folding, Sorting and Degradation;Chaperones and folding catalysts | 1.04% | 1.05% | 1.02% | 1.04% | 1.06% | 1.07% |
| **Genetic Information Processing** |  | | | | | |
| Folding, Sorting and Degradation;Protein export | 0.56% | 0.57% | 0.58% | 0.55% | 0.57% | 0.55% |
| Folding, Sorting and Degradation;RNA degradation | 0.45% | 0.45% | 0.45% | 0.46% | 0.46% | 0.45% |
| Folding, Sorting and Degradation;Sulfur relay system | 0.28% | 0.27% | 0.28% | 0.27% | 0.28% | 0.29% |
| Replication and Repair;Base excision repair | 0.43% | 0.44% | 0.44% | 0.43% | 0.44% | 0.43% |
| Replication and Repair;Chromosome | 1.52% | 1.52% | 1.55% | 1.50% | 1.53% | 1.50% |
| Replication and Repair;DNA repair and recombination proteins | 2.70% | 2.72% | 2.79% | 2.64% | 2.69% | 2.63% |
| Replication and Repair;DNA replication | 0.60% | 0.61% | 0.63% | 0.58% | 0.59% | 0.58% |
| Replication and Repair;DNA replication proteins | 1.10% | 1.11% | 1.16% | 1.07% | 1.08% | 1.06% |
| Replication and Repair;Homologous recombination | 0.86% | 0.86% | 0.88% | 0.82% | 0.84% | 0.81% |
| Replication and Repair;Mismatch repair | 0.75% | 0.76% | 0.78% | 0.72% | 0.72% | 0.71% |
| Replication and Repair;Non-homologous end-joining | 0.02% | 0.02% | 0.02% | 0.02% | 0.02% | 0.02% |
| Replication and Repair;Nucleotide excision repair | 0.34% | 0.35% | 0.36% | 0.33% | 0.34% | 0.32% |
| Transcription;RNA polymerase | 0.15% | 0.16% | 0.17% | 0.15% | 0.16% | 0.15% |
| Transcription;Transcription factors | 1.71% | 1.68% | 1.78% | 1.68% | 1.67% | 1.70% |
| Transcription;Transcription machinery | 0.86% | 0.90% | 0.88% | 0.89% | 0.86% | 0.85% |
| Translation;Aminoacyl-tRNA biosynthesis | 1.02% | 1.03% | 1.07% | 0.99% | 1.01% | 0.95% |
| Translation;Ribosome | 2.09% | 2.11% | 2.19% | 2.04% | 2.09% | 1.98% |
| Translation;Ribosome Biogenesis | 1.38% | 1.39% | 1.42% | 1.38% | 1.40% | 1.40% |
| Translation;RNA transport | 0.13% | 0.13% | 0.13% | 0.13% | 0.12% | 0.13% |
| Translation;Translation factors | 0.49% | 0.49% | 0.51% | 0.49% | 0.50% | 0.48% |
| **Metabolism;Amino Acid Metabolism** |  | | | | | |
| Alanine, aspartate and glutamate metabolism | 1.01% | 1.00% | 1.01% | 0.99% | 1.02% | 0.96% |
| Amino acid related enzymes | 1.35% | 1.35% | 1.37% | 1.33% | 1.36% | 1.31% |
| Arginine and proline metabolism | 1.13% | 1.12% | 1.10% | 1.12% | 1.09% | 1.09% |
| Cysteine and methionine metabolism | 0.95% | 0.96% | 0.97% | 0.96% | 0.96% | 0.94% |
| Glycine, serine and threonine metabolism | 0.82% | 0.82% | 0.79% | 0.83% | 0.81% | 0.82% |
| Histidine metabolism | 0.60% | 0.58% | 0.58% | 0.59% | 0.58% | 0.57% |
| Lysine biosynthesis | 0.77% | 0.77% | 0.78% | 0.74% | 0.73% | 0.73% |
| Lysine degradation | 0.19% | 0.18% | 0.17% | 0.21% | 0.20% | 0.21% |
| Phenylalanine metabolism | 0.23% | 0.23% | 0.21% | 0.24% | 0.23% | 0.23% |
| Phenylalanine, tyrosine and tryptophan biosynthesis | 0.77% | 0.77% | 0.77% | 0.76% | 0.74% | 0.75% |
| Tryptophan metabolism | 0.25% | 0.25% | 0.22% | 0.29% | 0.29% | 0.30% |
| Tyrosine metabolism | 0.40% | 0.38% | 0.39% | 0.39% | 0.40% | 0.40% |
| Valine, leucine and isoleucine biosynthesis | 0.71% | 0.69% | 0.69% | 0.70% | 0.67% | 0.69% |
| Valine, leucine and isoleucine degradation | 0.33% | 0.33% | 0.30% | 0.39% | 0.37% | 0.38% |
| **Metabolism; Biosynthesis of Other Secondary Metabolites** |  | | | | | |
| beta-Lactam resistance | 0.03% | 0.04% | 0.03% | 0.04% | 0.04% | 0.04% |
| Butirosin and neomycin biosynthesis | 0.06% | 0.07% | 0.07% | 0.06% | 0.07% | 0.06% |
| Caffeine metabolism | 0.00% | 0.00% | 0.00% | 0.00% | 0.00% | 0.00% |
| Flavone and flavonol biosynthesis | 0.00% | 0.00% | 0.00% | 0.00% | 0.00% | 0.00% |
| Flavonoid biosynthesis | 0.01% | 0.01% | 0.01% | 0.00% | 0.00% | 0.00% |
| Isoflavonoid biosynthesis | 0.00% | 0.00% | 0.00% | 0.00% | 0.00% | 0.00% |
| Isoquinoline alkaloid biosynthesis | 0.05% | 0.05% | 0.05% | 0.05% | 0.05% | 0.05% |
| Novobiocin biosynthesis | 0.13% | 0.13% | 0.13% | 0.13% | 0.13% | 0.13% |
| Penicillin and cephalosporin biosynthesis | 0.04% | 0.04% | 0.04% | 0.05% | 0.05% | 0.05% |
| Phenylpropanoid biosynthesis | 0.11% | 0.13% | 0.11% | 0.13% | 0.14% | 0.12% |
| Stilbenoid, diarylheptanoid and gingerol biosynthesis | 0.00% | 0.00% | 0.00% | 0.00% | 0.00% | 0.00% |
| Streptomycin biosynthesis | 0.30% | 0.31% | 0.29% | 0.30% | 0.30% | 0.29% |
| Tropane, piperidine and pyridine alkaloid biosynthesis | 0.12% | 0.12% | 0.11% | 0.12% | 0.12% | 0.12% |
| **Metabolism; Carbohydrate Metabolism** |  | | | | | |
| Amino sugar and nucleotide sugar metabolism | 1.34% | 1.36% | 1.39% | 1.34% | 1.37% | 1.31% |
| Ascorbate and aldarate metabolism | 0.15% | 0.15% | 0.16% | 0.16% | 0.16% | 0.16% |
| Butanoate metabolism | 0.73% | 0.71% | 0.71% | 0.74% | 0.75% | 0.74% |
| C5-Branched dibasic acid metabolism | 0.30% | 0.30% | 0.29% | 0.30% | 0.27% | 0.30% |
| Citrate cycle (TCA cycle) | 0.70% | 0.69% | 0.64% | 0.70% | 0.67% | 0.70% |
| Fructose and mannose metabolism | 0.92% | 0.92% | 0.97% | 0.87% | 0.91% | 0.86% |
| Galactose metabolism | 0.70% | 0.70% | 0.73% | 0.67% | 0.69% | 0.64% |
| Glycolysis / Gluconeogenesis | 1.13% | 1.13% | 1.15% | 1.08% | 1.10% | 1.07% |
| Glyoxylate and dicarboxylate metabolism | 0.57% | 0.56% | 0.54% | 0.57% | 0.57% | 0.58% |
| Inositol phosphate metabolism | 0.13% | 0.13% | 0.12% | 0.14% | 0.14% | 0.14% |
| Pentose and glucuronate interconversions | 0.54% | 0.55% | 0.54% | 0.52% | 0.53% | 0.50% |
| Pentose phosphate pathway | 0.90% | 0.91% | 0.92% | 0.87% | 0.88% | 0.86% |
| Propanoate metabolism | 0.61% | 0.60% | 0.59% | 0.61% | 0.61% | 0.62% |
| Pyruvate metabolism | 1.10% | 1.08% | 1.10% | 1.06% | 1.07% | 1.05% |
| Starch and sucrose metabolism | 0.86% | 0.89% | 0.91% | 0.85% | 0.90% | 0.81% |
| Carbon fixation in photosynthetic organisms | 0.60% | 0.60% | 0.61% | 0.57% | 0.58% | 0.56% |
| Carbon fixation pathways in prokaryotes | 1.02% | 1.01% | 0.98% | 1.00% | 0.98% | 1.00% |
| **Metabolism; Energy Metabolism** |  | | | | | |
| Methane metabolism | 1.18% | 1.16% | 1.18% | 1.15% | 1.09% | 1.14% |
| Nitrogen metabolism | 0.71% | 0.71% | 0.71% | 0.72% | 0.72% | 0.73% |
| Oxidative phosphorylation | 1.19% | 1.21% | 1.14% | 1.22% | 1.21% | 1.23% |
| Photosynthesis | 0.30% | 0.31% | 0.30% | 0.30% | 0.31% | 0.29% |
| Photosynthesis - antenna proteins | 0.00% | 0.00% | 0.00% | 0.00% | 0.00% | 0.00% |
| Photosynthesis proteins | 0.31% | 0.31% | 0.31% | 0.30% | 0.32% | 0.30% |
| Sulfur metabolism | 0.30% | 0.31% | 0.29% | 0.32% | 0.32% | 0.32% |
| **Metabolism; Enzyme Families** |  | | | | | |
| Cytochrome P450 | 0.00% | 0.00% | 0.00% | 0.00% | 0.00% | 0.00% |
| Peptidases | 1.79% | 1.81% | 1.82% | 1.78% | 1.81% | 1.76% |
| Protein kinases | 0.36% | 0.36% | 0.34% | 0.37% | 0.36% | 0.39% |
| **Metabolism; Glycan Biosynthesis and Metabolism** |  | | | | | |
| Glycosaminoglycan biosynthesis - chondroitin sulfate | 0.00% | 0.00% | 0.00% | 0.00% | 0.00% | 0.00% |
| Glycosaminoglycan degradation | 0.08% | 0.07% | 0.06% | 0.08% | 0.06% | 0.07% |
| Glycosphingolipid biosynthesis - ganglio series | 0.06% | 0.06% | 0.04% | 0.06% | 0.05% | 0.06% |
| Glycosphingolipid biosynthesis - globo series | 0.11% | 0.11% | 0.10% | 0.11% | 0.10% | 0.11% |
| Glycosphingolipid biosynthesis - lacto and neolacto series | 0.00% | 0.00% | 0.00% | 0.00% | 0.00% | 0.00% |
| Glycosyltransferases | 0.37% | 0.36% | 0.36% | 0.36% | 0.37% | 0.38% |
| Lipopolysaccharide biosynthesis | 0.32% | 0.31% | 0.27% | 0.32% | 0.31% | 0.35% |
| Lipopolysaccharide biosynthesis proteins | 0.48% | 0.45% | 0.41% | 0.48% | 0.47% | 0.52% |
| N-Glycan biosynthesis | 0.02% | 0.02% | 0.02% | 0.02% | 0.02% | 0.02% |
| Other glycan degradation | 0.27% | 0.27% | 0.23% | 0.27% | 0.24% | 0.24% |
| Peptidoglycan biosynthesis | 0.74% | 0.75% | 0.78% | 0.73% | 0.75% | 0.72% |
| Various types of N-glycan biosynthesis | 0.00% | 0.00% | 0.00% | 0.00% | 0.00% | 0.00% |
| **Metabolism; Lipid Metabolism** |  | | | | | |
| alpha-Linolenic acid metabolism | 0.02% | 0.02% | 0.02% | 0.03% | 0.03% | 0.03% |
| Arachidonic acid metabolism | 0.05% | 0.05% | 0.04% | 0.06% | 0.06% | 0.06% |
| Biosynthesis of unsaturated fatty acids | 0.19% | 0.19% | 0.18% | 0.20% | 0.20% | 0.22% |
| Ether lipid metabolism | 0.01% | 0.01% | 0.01% | 0.01% | 0.01% | 0.01% |
| Fatty acid biosynthesis | 0.51% | 0.51% | 0.51% | 0.50% | 0.50% | 0.50% |
| Fatty acid elongation in mitochondria | 0.00% | 0.00% | 0.00% | 0.00% | 0.00% | 0.00% |
| Fatty acid metabolism | 0.33% | 0.31% | 0.30% | 0.35% | 0.34% | 0.35% |
| Glycerolipid metabolism | 0.41% | 0.40% | 0.43% | 0.38% | 0.39% | 0.37% |
| Glycerophospholipid metabolism | 0.56% | 0.55% | 0.57% | 0.55% | 0.56% | 0.56% |
| Linoleic acid metabolism | 0.06% | 0.07% | 0.06% | 0.06% | 0.07% | 0.06% |
| Lipid biosynthesis proteins | 0.63% | 0.64% | 0.62% | 0.64% | 0.64% | 0.64% |
| Primary bile acid biosynthesis | 0.03% | 0.03% | 0.03% | 0.03% | 0.03% | 0.03% |
| Secondary bile acid biosynthesis | 0.03% | 0.03% | 0.03% | 0.02% | 0.03% | 0.02% |
| Sphingolipid metabolism | 0.18% | 0.18% | 0.17% | 0.18% | 0.16% | 0.16% |
| Steroid biosynthesis | 0.00% | 0.00% | 0.00% | 0.00% | 0.00% | 0.00% |
| Steroid hormone biosynthesis | 0.01% | 0.02% | 0.01% | 0.02% | 0.01% | 0.01% |
| Synthesis and degradation of ketone bodies | 0.07% | 0.07% | 0.06% | 0.08% | 0.08% | 0.08% |
| Metabolism;Metabolism of Cofactors and Vitamins |  | | | | | |
| Biotin metabolism | 0.16% | 0.15% | 0.15% | 0.16% | 0.15% | 0.16% |
| Folate biosynthesis | 0.42% | 0.42% | 0.41% | 0.42% | 0.43% | 0.43% |
| Lipoic acid metabolism | 0.05% | 0.05% | 0.05% | 0.06% | 0.05% | 0.06% |
| Nicotinate and nicotinamide metabolism | 0.44% | 0.43% | 0.44% | 0.43% | 0.44% | 0.43% |
| One carbon pool by folate | 0.56% | 0.56% | 0.56% | 0.56% | 0.56% | 0.54% |
| Pantothenate and CoA biosynthesis | 0.57% | 0.56% | 0.57% | 0.57% | 0.55% | 0.55% |
| Porphyrin and chlorophyll metabolism | 0.81% | 0.81% | 0.81% | 0.82% | 0.75% | 0.80% |
| Retinol metabolism | 0.05% | 0.05% | 0.04% | 0.05% | 0.05% | 0.05% |
| Riboflavin metabolism | 0.24% | 0.24% | 0.22% | 0.24% | 0.24% | 0.25% |
| Thiamine metabolism | 0.43% | 0.42% | 0.43% | 0.40% | 0.40% | 0.40% |
| Ubiquinone and other terpenoid-quinone biosynthesis | 0.25% | 0.25% | 0.23% | 0.26% | 0.27% | 0.29% |
| Vitamin B6 metabolism | 0.19% | 0.19% | 0.19% | 0.19% | 0.19% | 0.19% |
| **Metabolism; Metabolism of Other Amino Acids** |  | | | | | |
| beta-Alanine metabolism | 0.22% | 0.20% | 0.20% | 0.23% | 0.22% | 0.23% |
| Cyanoamino acid metabolism | 0.25% | 0.26% | 0.24% | 0.27% | 0.28% | 0.24% |
| D-Alanine metabolism | 0.10% | 0.10% | 0.11% | 0.10% | 0.11% | 0.10% |
| D-Arginine and D-ornithine metabolism | 0.00% | 0.00% | 0.00% | 0.00% | 0.00% | 0.00% |
| D-Glutamine and D-glutamate metabolism | 0.13% | 0.13% | 0.13% | 0.13% | 0.13% | 0.12% |
| Glutathione metabolism | 0.33% | 0.33% | 0.31% | 0.36% | 0.38% | 0.38% |
| Phosphonate and phosphinate metabolism | 0.06% | 0.06% | 0.06% | 0.06% | 0.06% | 0.06% |
| Selenocompound metabolism | 0.37% | 0.38% | 0.38% | 0.38% | 0.40% | 0.38% |
| Taurine and hypotaurine metabolism | 0.11% | 0.11% | 0.10% | 0.11% | 0.11% | 0.11% |
| **Metabolism; Metabolism of Terpenoids and Polyketides** |  | | | | | |
| Biosynthesis of ansamycins | 0.10% | 0.10% | 0.10% | 0.09% | 0.09% | 0.09% |
| Biosynthesis of siderophore group nonribosomal peptides | 0.04% | 0.04% | 0.04% | 0.04% | 0.05% | 0.05% |
| Biosynthesis of type II polyketide products | 0.00% | 0.00% | 0.00% | 0.00% | 0.00% | 0.00% |
| Biosynthesis of vancomycin group antibiotics | 0.06% | 0.06% | 0.06% | 0.06% | 0.06% | 0.06% |
| Carotenoid biosynthesis | 0.00% | 0.00% | 0.01% | 0.00% | 0.00% | 0.00% |
| Geraniol degradation | 0.11% | 0.10% | 0.09% | 0.13% | 0.13% | 0.14% |
| Limonene and pinene degradation | 0.13% | 0.12% | 0.12% | 0.13% | 0.13% | 0.14% |
| Polyketide sugar unit biosynthesis | 0.19% | 0.19% | 0.18% | 0.19% | 0.18% | 0.18% |
| Prenyltransferases | 0.28% | 0.28% | 0.28% | 0.28% | 0.28% | 0.27% |
| Terpenoid backbone biosynthesis | 0.51% | 0.52% | 0.53% | 0.50% | 0.51% | 0.48% |
| Tetracycline biosynthesis | 0.16% | 0.16% | 0.17% | 0.15% | 0.15% | 0.16% |
| Zeatin biosynthesis | 0.04% | 0.05% | 0.04% | 0.04% | 0.04% | 0.04% |
| **Metabolism; Nucleotide Metabolism** |  | | | | | |
| Purine metabolism | 2.18% | 2.19% | 2.23% | 2.14% | 2.20% | 2.15% |
| Pyrimidine metabolism | 1.70% | 1.71% | 1.76% | 1.65% | 1.68% | 1.63% |
| **Metabolism; Xenobiotics Biodegradation and Metabolism** |  | | | | | |
| 1,1,1-Trichloro-2,2-bis(4-chlorophenyl)ethane (DDT) degradation | 0.00% | 0.00% | 0.00% | 0.00% | 0.00% | 0.00% |
| Aminobenzoate degradation | 0.18% | 0.18% | 0.16% | 0.21% | 0.20% | 0.20% |
| Atrazine degradation | 0.01% | 0.01% | 0.01% | 0.01% | 0.01% | 0.01% |
| Benzoate degradation | 0.25% | 0.24% | 0.24% | 0.26% | 0.25% | 0.23% |
| Bisphenol degradation | 0.07% | 0.07% | 0.07% | 0.07% | 0.07% | 0.06% |
| Caprolactam degradation | 0.08% | 0.07% | 0.07% | 0.09% | 0.09% | 0.09% |
| Chloroalkane and chloroalkene degradation | 0.21% | 0.21% | 0.21% | 0.21% | 0.22% | 0.20% |
| Chlorocyclohexane and chlorobenzene degradation | 0.04% | 0.04% | 0.03% | 0.04% | 0.04% | 0.05% |
| Dioxin degradation | 0.06% | 0.06% | 0.07% | 0.05% | 0.06% | 0.05% |
| Drug metabolism - cytochrome P450 | 0.10% | 0.10% | 0.09% | 0.11% | 0.12% | 0.12% |
| Drug metabolism - other enzymes | 0.29% | 0.30% | 0.30% | 0.29% | 0.29% | 0.28% |
| Ethylbenzene degradation | 0.05% | 0.05% | 0.05% | 0.05% | 0.05% | 0.05% |
| Fluorobenzoate degradation | 0.01% | 0.01% | 0.01% | 0.01% | 0.01% | 0.01% |
| Metabolism of xenobiotics by cytochrome P450 | 0.10% | 0.10% | 0.09% | 0.11% | 0.12% | 0.12% |
| Naphthalene degradation | 0.18% | 0.18% | 0.18% | 0.17% | 0.18% | 0.17% |
| Nitrotoluene degradation | 0.08% | 0.08% | 0.08% | 0.08% | 0.07% | 0.08% |
| Polycyclic aromatic hydrocarbon degradation | 0.08% | 0.07% | 0.08% | 0.07% | 0.07% | 0.06% |
| Styrene degradation | 0.04% | 0.04% | 0.04% | 0.05% | 0.05% | 0.05% |
| Toluene degradation | 0.12% | 0.12% | 0.11% | 0.13% | 0.13% | 0.14% |
| Xylene degradation | 0.06% | 0.05% | 0.06% | 0.05% | 0.05% | 0.04% |
| **Unclassified** |  | | | | | |
| Unclassified;Cellular Processes and Signaling; Cell division | 0.07% | 0.07% | 0.07% | 0.08% | 0.07% | 0.08% |
| Unclassified;Cellular Processes and Signaling; Cell motility and secretion | 0.23% | 0.24% | 0.21% | 0.25% | 0.25% | 0.26% |
| Unclassified;Cellular Processes and Signaling; Electron transfer carriers | 0.04% | 0.04% | 0.04% | 0.04% | 0.04% | 0.05% |
| Unclassified;Cellular Processes and Signaling; Germination | 0.02% | 0.03% | 0.03% | 0.02% | 0.02% | 0.02% |
| Unclassified;Cellular Processes; Inorganic ion transport and metabolism | 0.30% | 0.30% | 0.28% | 0.32% | 0.32% | 0.34% |
| Unclassified;Cellular Processes; Membrane and intracellular structural molecules | 0.71% | 0.72% | 0.63% | 0.77% | 0.77% | 0.80% |
| Unclassified;Cellular Processes and Signaling; Other ion-coupled transporters | 1.35% | 1.32% | 1.28% | 1.32% | 1.31% | 1.36% |
| Unclassified;Cellular Processes and Signaling; Other transporters | 0.32% | 0.32% | 0.32% | 0.32% | 0.33% | 0.34% |
| Unclassified;Cellular Processes and Signaling; Pores ion channels | 0.50% | 0.51% | 0.45% | 0.55% | 0.55% | 0.58% |
| Unclassified;Cellular Processes and Signaling; Signal transduction mechanisms | 0.48% | 0.47% | 0.50% | 0.48% | 0.50% | 0.50% |
| Unclassified;Cellular Processes and Signaling; Sporulation | 0.47% | 0.48% | 0.54% | 0.39% | 0.33% | 0.34% |
| Unclassified; Protein folding and associated processing | 0.65% | 0.65% | 0.63% | 0.65% | 0.65% | 0.67% |
| Unclassified;;Replication, recombination and repair proteins | 0.78% | 0.77% | 0.82% | 0.77% | 0.79% | 0.80% |
| Unclassified; Genetic Information Processing; Restriction enzyme | 0.16% | 0.17% | 0.15% | 0.15% | 0.14% | 0.15% |
| Unclassified; Genetic Information Processing; Transcription related proteins | 0.01% | 0.01% | 0.01% | 0.01% | 0.01% | 0.02% |
| Unclassified; Genetic Information Processing; Translation proteins | 0.88% | 0.88% | 0.91% | 0.87% | 0.88% | 0.87% |
| Unclassified; Metabolism; Amino acid metabolism | 0.22% | 0.21% | 0.21% | 0.22% | 0.20% | 0.21% |
| Unclassified; Biosynthesis and biodegradation of secondary metabolites | 0.07% | 0.07% | 0.06% | 0.07% | 0.06% | 0.07% |
| Unclassified; Metabolism; Carbohydrate metabolism | 0.15% | 0.16% | 0.16% | 0.14% | 0.14% | 0.13% |
| Unclassified; Metabolism; Energy metabolism | 0.88% | 0.85% | 0.85% | 0.88% | 0.83% | 0.87% |
| Unclassified; Metabolism; Glycan biosynthesis and metabolism | 0.06% | 0.06% | 0.06% | 0.07% | 0.07% | 0.08% |
| Unclassified; Metabolism; Lipid metabolism | 0.12% | 0.12% | 0.13% | 0.12% | 0.12% | 0.12% |
| Unclassified; Metabolism; Metabolism of cofactors and vitamins | 0.14% | 0.14% | 0.14% | 0.15% | 0.15% | 0.15% |
| Unclassified; Metabolism; Nucleotide metabolism | 0.06% | 0.05% | 0.05% | 0.05% | 0.05% | 0.06% |
| Unclassified; Metabolism; Others | 0.93% | 0.94% | 0.94% | 0.94% | 0.92% | 0.94% |
| Unclassified; Poorly Characterized; Function unknown | 1.52% | 1.49% | 1.49% | 1.55% | 1.60% | 1.63% |
